# Stably expressed APOBEC3H forms a barrier for cross-species transmission of simian immunodeficiency virus of chimpanzee to humans

**DOI:** 10.1371/journal.ppat.1006746

**Published:** 2017-12-21

**Authors:** Zeli Zhang, Qinyong Gu, Marc de Manuel Montero, Ignacio G. Bravo, Tomas Marques-Bonet, Dieter Häussinger, Carsten Münk

**Affiliations:** 1 Clinic for Gastroenterology, Hepatology, and Infectiology, Medical Faculty, Heinrich-Heine-Universität Düsseldorf, Düsseldorf, Germany; 2 Institut Biologia Evolutiva (Universitat Pompeu Fabra/CSIC) ICREA, Barcelona, Spain; 3 Laboratory MIVEGEC, UMR CNRS 5290, UM, Montpellier, France; University of Illinois at Chicago College of Medicine, UNITED STATES

## Abstract

APOBEC3s (A3s) are potent restriction factors of human immunodeficiency virus type 1/simian immunodeficiency viruses (HIV-1/SIV), and can repress cross-species transmissions of lentiviruses. HIV-1 originated from a zoonotic infection of SIV of chimpanzee (SIVcpz) to humans. However, the impact of human A3s on the replication of SIVcpz remains unclear. By using novel SIVcpz reporter viruses, we identified that human APOBEC3B (A3B) and APOBEC3H (A3H) haplotype II strongly reduced the infectivity of SIVcpz, because both of them are resistant to SIVcpz Vifs. We further demonstrated that human A3H inhibited SIVcpz by deaminase dependent as well independent mechanisms. In addition, other stably expressed human A3H haplotypes and splice variants showed strong antiviral activity against SIVcpz. Moreover, most SIV and HIV lineage Vif proteins could degrade chimpanzee A3H, but no Vifs from SIVcpz and SIV of gorilla (SIVgor) lineages antagonized human A3H haplotype II. Expression of human A3H hapII in human T cells efficiently blocked the spreading replication of SIVcpz. The spreading replication of SIVcpz was also restricted by stable A3H in human PBMCs. Thus, we speculate that stably expressed human A3H protects humans against the cross-species transmission of SIVcpz and that SIVcpz spillover to humans may have started in individuals that harbor haplotypes of unstable A3H proteins.

## Author summary

Cellular cytidine deaminases of the APOBEC3 (A3) family are potent restriction factors that are able to inhibit retroviruses, this A3 restriction is counteracted by lentivirus Vif proteins. Human APOBEC3H (A3H) represents the most evolutionarily divergent A3 gene; it includes seven haplotypes and several splice variants. The polymorphism of human A3H has relevance for HIV-1 infection and AIDS progression. HIV-1 originated from cross-transmission of SIVcpz to humans. However, little is known about how human A3s affect the replication or transmission of SIVcpz. In this study, we comprehensively analyzed the anti-SIVcpz activity of chimpanzee and human A3s. Human A3H haplotype II was identified as strong inhibitor against SIVcpz regardless of Vif. In addition, other stably expressed human A3H haplotypes and splice variants showed strong antiviral activity against SIVcpz. Moreover, based on the recent Great Ape Genome Project, we found that the polymorphism of chimpanzee A3H is lower compared with the diversity of human A3H. And chimpanzee A3H haplotypes identified in this study showed similar anti-SIVcpz activity and Vif sensitivity. Our results provide a model that stably expressed human A3H protects humans against the cross-species transmission of SIVcpz and that SIVcpz spillover to humans may have started in individuals that harbor haplotypes of unstable A3H proteins.

## Introduction

Simian immunodeficiency virus (SIV) naturally infects many species of African Old-World monkeys, such as African green monkeys, mandrills and red-capped mangabey [1,2]. However, these viruses appear to be nonpathogenic in their natural hosts [2,3]. Chimpanzees (cpz), which are the evolutionarily closest extant primate to *Homo sapiens*, are infected by SIVcpz [4]. The common chimpanzee includes four subspecies, only two of which, *Pan troglodytes troglodytes* (*Ptt*) and *Pan troglodytes schweinfurthii* (*Pts*), are infected by SIVcpz (SIVcpz*Ptt* and SIVcpz*Pts*, respectively) [4]. Genome analysis of SIVcpz indicates that SIVcpz originates from the cross-species transmission and recombination of three different SIV strains: SIVrcm from the red-capped mangabey (rcm), SIVgsn/mus/mon from the greater-spot-nosed (gsn), mustached (mus), and mona monkeys (mon), respectively, and a currently unidentified SIV [5–7]. SIVcpz*Pts* is thought to be the origin of SIVcpz*Ptt* after intra-chimpanzee transmission [5].

SIVcpz is of particular interest because it is the ancestor of human immunodeficiency virus (HIV)-1. HIV-1 M and N groups originated from zoonotic transmission of SIVcpz*Ptt* from west-central Africa [8,9]. Additionally, recent studies indicate that SIVgor from gorillas (gor) is the origin of HIV-1 groups O and P [10,11]. The HIV-1 M group is the pandemic virus, whereas viruses of groups N and P are only found in a few infected individuals [12,13]. The HIV-1 O group is mainly distributed in west-central Africa and has a low prevalence rate (less than 1% of global HIV-1 infections) [14,15]. The other HIV lentivirus, HIV-2, resulted from cross-species transmission of SIV from the sooty mangabey monkey (SIVsmm) [14].

Human intrinsic cellular antiviral factors may have direct relevance for the zoonotic infection of humans and the human-to-human spread of SIVs. Several restriction factors have been identified that repress lentiviral replication [16–18]. The family of human APOBEC3 (A3) restriction factors is formed by seven different proteins, A3A–D and A3F–H. Virion encapsidated APOBEC3D (A3D), A3F, A3G, and A3H inhibit HIV-1 that lacks the gene *vif* (HIV-1Δ*vif*) by deaminating cytidines in the viral single-stranded DNA that is generated during reverse transcription, thereby introducing G-to-A hypermutations in the coding strand [19]. To achieve productive infections, lentiviral Vif proteins directly interact with A3s and recruit them to an E3 ubiquitin ligase complex to induce A3 degradation by the proteasome [20–22]. Several studies have investigated how A3G serves as a barrier for cross-species transmission of lentiviruses [23–25]. Human A3H represents the most evolutionarily divergent A3 gene; it includes seven haplotypes and several splice variants [26–28]. The protein stability of human A3H is one determinant of its antiviral activity [29–31]. The human A3H haplotype (hap) II, in contrast to A3G, is only sensitive to specific HIV-1 Vifs and adaptation of HIV-1 Vif to A3H hapII has been described [32–35]. Thus, the polymorphism of human A3H has relevance for HIV-1 infection and AIDS progression [36,37].

To investigate how human A3s may affect the replication of SIVcpz, we generated novel luciferase reporter viruses based on two SIVcpz strains (SIVcpz*Pts*TAN1 and SIVcpz*Ptt*MB897). This system revealed that SIVcpz transmission to humans may have been significantly affected by the presence of stable A3H.

## Results

### Characterization of nanoluciferase reporter viruses for SIVcpz

**Fig 1:**
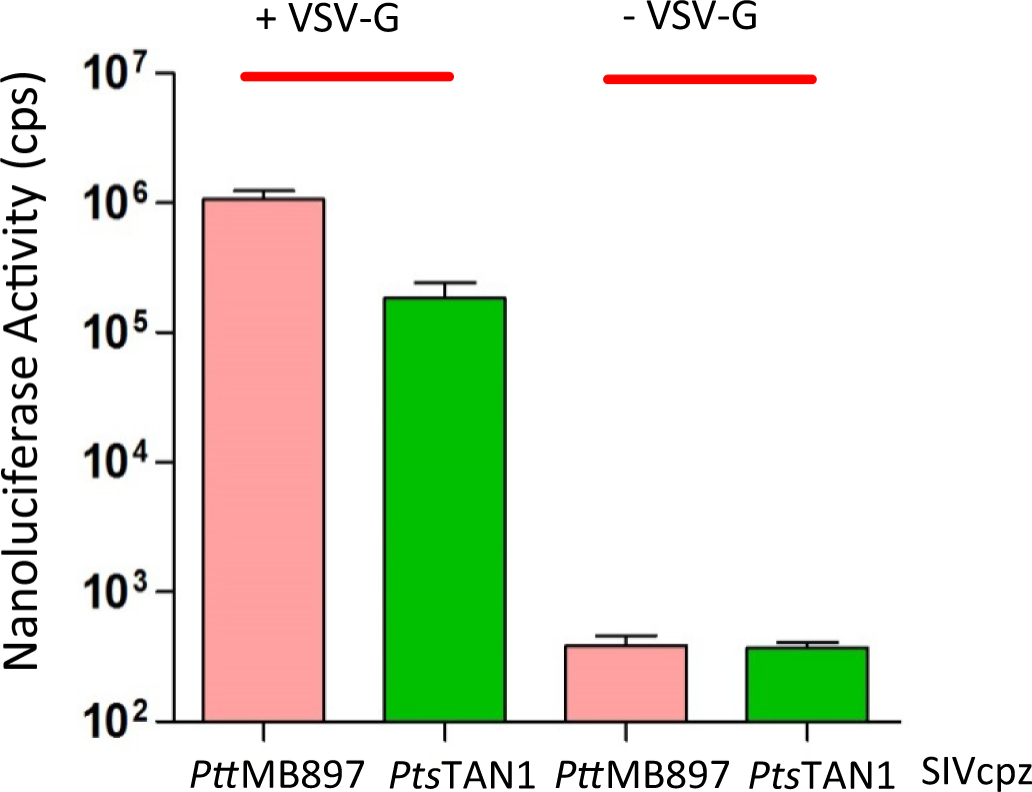
Single round infection assay of SIVcpz reporter viruses. SIVcpz-NLuc was produced in 293T cells in the presence (+) or absence (-) of VSV-G expression plasmid. Virions that had 20 pg reverse transcription activity were used for infection of 293T cells, viral infectivity was determined by quantification of nanoluciferase activity in lysates of infected cells, cps counts per second.

To test SIVcpz, we first generated SIVcpz nanoluciferase (*NLuc*) reporter viruses using two SIV strains (SIVcpz*Ptt*MB897 and SIVcpz*Pts*TAN1; S1 Fig). SIVcpz*Ptt*MB897 was isolated from wild chimpanzee (*Pan troglodytes troglodytes*) in southern Cameroon in 2007 [9,38], and this strain is regarded as the ancestor of the pandemic HIV-1 M group [14]. SIVcpz*Pts*TAN1 was derived from chimpanzee subspecies *Pan troglodytes schweinfurthii*, and this strain does not cause sustained infections of humans [39]. The SIVcpz reporter constructs were generated by replacing most of the *nef* gene with *NLuc*. Additionally, the *vif* gene of SIVcpz was inactivated (S1 Fig). SIVcpz-NLuc reporter viruses pseudotyped with the glycoprotein of the vesicular stomatitis virus (VSV-G) were produced by plasmid transfection of 293T cells. When infected with the VSV-G pseudotyped viruses, SIVcpz*Ptt*MB897-NLuc and SIVcpz*Pts*TAN1-NLuc, 293T cells showed high luciferase counts while very low nanoluciferase activity was detected when the viruses were not VSV-G pseudotyped (Fig 1). The luciferase activity of SIVcpz*Pts*TAN1-NLuc was around 10-fold less than SIVcpz*Ptt*MB897-NLuc, even when equal amounts of virions normalized for reverse transcription activity were used for infection (Fig 1). Thus, these two novel SIVcpz reporter viruses transmitted the luciferase enzyme activity via glycoprotein-dependent infection.

### Chimpanzee APOBEC3s have antiviral activity against diverse SIVs and are antagonized by SIVcpz Vif

**Fig 2:**
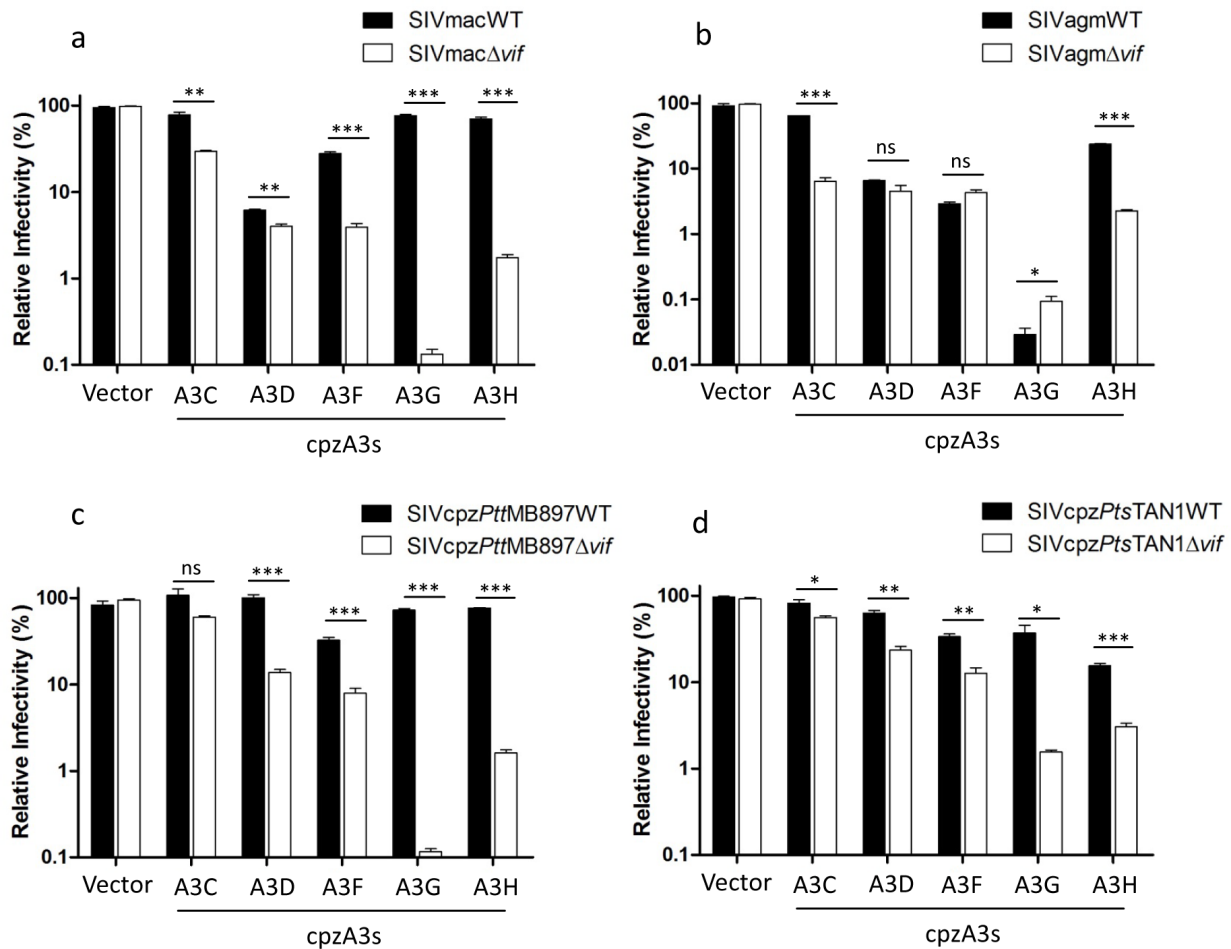
Chimpanzee APOBEC3s (cpzA3s) inhibit SIVs. (**a, b**) SIVmac or SIVagm wild type or delta *vif* reporter viruses were produced in 293T cells in the presence of cpzA3s. pcDNA3.1(+) was used as control (vector) for cpzA3s. Two days post-transfection, normalized amounts of viruses were used to infect 293T cells, firefly luciferase (relative light units-RLU) was measured two days post-infection. (**c, d**) SIVcpz*Ptt*MB897 or SIVcpz*Pts*TAN1 wild type or delta *vif* reporter viruses were produced in 293T cells in the presence of cpzA3s. pcDNA3.1(+) was used as control (vector) for cpzA3s. Two days post-transfection, normalized amounts of viruses were used to infect 293T cells. Two days post-infection, 293T cells were carefully washed once with PBS, and nanoluciferase (relative light units-RLU) was measured, relative infectivity was shown. Values are means plus standard deviations (error bars) of a representative experiment performed in triplicate. Asterisks represent statistically significant differences: P value < 0.001 extremely significant (***), 0.001 to 0.01 very significant (**), 0.01 to 0.05 significant (*), >0.05 not significant (ns).

Four SIV-luciferase reporter viruses based on SIV of macaques (SIVmac), African green monkeys (SIVagm) and chimpanzees (SIVcpz*Pts*, and SIVcpz*Ptt*) were used to investigate the antiviral activity of chimpanzee A3s. We found that cpzA3C, D, F, G, and H reduced the infectivity of SIVmacΔ*vif* (Fig 2A). SIVmac Vif fully antagonized restrictions of cpzA3C, G, and H, and to a large extent overcame cpzA3F, but it did not inhibit the restriction of cpzA3D (Fig 2A). Chimpanzee A3s showed a similar restriction pattern against SIVagmΔ*vif*, but SIVagm Vif only abolished the restriction of cpzA3C and partly inhibited the restriction of cpzA3H. Even in the presence of SIVagm Vif, cpzA3D, F, and G significantly reduced the infectivity of SIVagm (Fig 2A). The expression of the cpzA3C, F, G, and H was detectable by immunoblotting using HA-tagged specific antibodies, while cpzA3D was not detectable using our immunoblotting system (S2A and S2C Fig).

In the absence of Vifs, cpzA3C reduced the infectivity of SIVmac and SIVagm by 5–10 fold and weakly inhibited SIVcpz*Ptt*MB897 and SIVcpz*Pts*TAN1 by 1–2 fold (Fig 2C and 2D). cpzA3D, F, and H inhibited SIVcpzΔ*vif* by 10–15-fold, while cpzA3G reduced the infectivity of both SIVcpz*Ptt*MB897Δ*vif* and SIVcpz*Pts*TAN1Δ*vif* to an even greater extent (Fig 2C and 2D). SIVcpz*Ptt*MB897 and SIVcpz*Pts*TAN1 Vifs are able to counteract all cpzA3s, but not all in cases to the same level, e.g. cpzA3F, the full viral infectivity (vector control) was restored, consistent with previous study [40] (Fig 2C and 2D). Taken together, these data indicate that chimpanzee A3s, such as cpzA3D and cpzA3G, can protect chimpanzees from infection with SIVs of rhesus macaques and African green monkeys.

### Human APOBEC3H haplotype II strongly inhibits SIVcpz infectivity and is resistant to SIVcpz Vif

**Fig 3:**
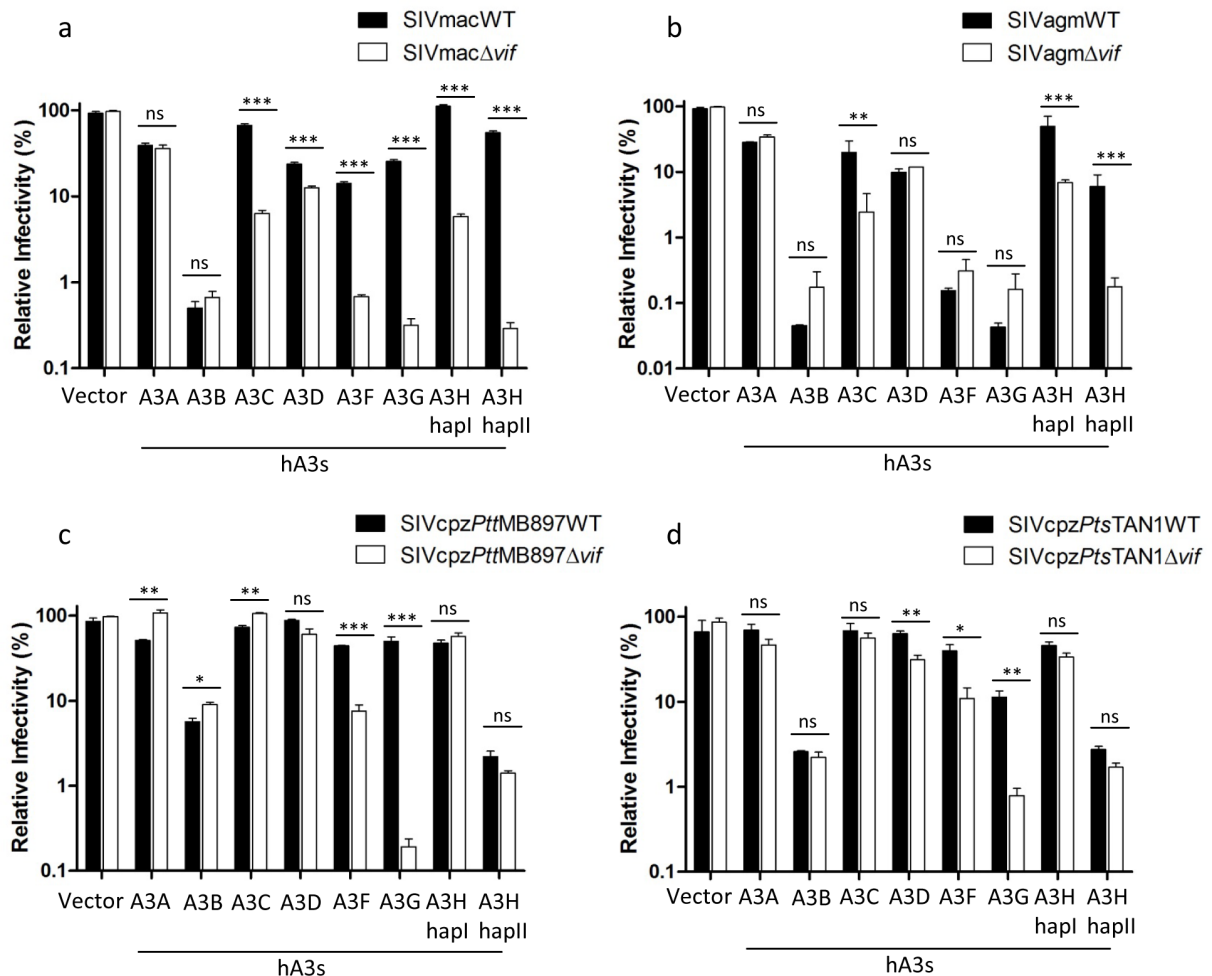
Human APOBEC3s (hA3s) inhibit SIVs. (**a, b**) SIVmac or SIVagm wild type or delta *vif* reporter viruses were produced in 293T cells in the presence of hA3s. PTR600 empty vector was used as control (vector) for hA3s. Two days post-transfection, normalized amounts of viruses were used to infect 293T cells, firefly luciferase (relative light units-RLU) was measured two days post-infection. (**c, d**) SIVcpz*Ptt*MB897 or SIVcpz*Pts*TAN1 wild type or delta *vif* reporter viruses were produced in 293T cells in the presence of hA3s. PTR600 empty vector was used as control (vector) for hA3s. Two days post-transfection, normalized amounts of viruses were used to infect 293T cells. Two days post-infection, 293T cells were carefully washed once with PBS, and nanoluciferase (relative light units-RLU) was measured. The relative infectivity was shown. Values are means plus standard deviations (error bars) of a representative experiment performed in triplicate. Asterisks represent statistically significant differences: P value < 0.001 extremely significant (***), 0.001 to 0.01 very significant (**), 0.01 to 0.05 significant (*), >0.05 not significant (ns).

Our data and a previous study indicate that chimpanzee A3s, especially cpzA3D, play an important role as a barrier to cross-species transmission of SIVs from monkeys to chimpanzees (Fig 2A and 2B) [40]. Next, we asked whether human A3s (hA3s) form a barrier to SIVcpz infection of humans. Thus, we analyzed the anti-SIV activity of human A3s by using the four SIV reporter systems. Similar to chimpanzee A3s, hA3C, D, F, G, and H (including hapI and hapII) inhibited SIVmacΔ*vif* and SIVagmΔ*vif* infections, and SIVmac Vif abolished most of these restrictions but was only weakly active against hA3D (Fig 3A). SIVagm Vif only significantly overcame the restriction of hA3C, hA3H hapI, and hA3H hapII (Fig 3B). hA3D, hA3F and hA3G displayed resistance to SIVagm Vif counteraction, indicating that these three factors may protect humans against infection by SIVagm (Fig 3B). Consistent with a previous study, hA3B strongly reduced the infectivity of SIVmac and SIVagm regardless of Vif [41]. However, hA3A showed only a low-level inhibition of SIVmac and SIVagm and this restriction was resistant to both SIVmac and SIVagm Vifs (Fig 3A and 3B). The expression of hA3s in transfected 293T cells was detected by immunoblotting (S2B and S2C Fig). In the absence of Vif, hA3D, F, and G reduced the infectivity of SIVcpz, while Vif proteins from both SIVcpz*Ptt*MB897 and SIVcpz*Pts*TAN1 antagonized these hA3s (Fig 3C and 3D). In contrast to the experiments with SIVmacΔ*vif* and SIVagmΔ*vif*, no antiviral activity of hA3C was seen against SIVcpzΔ*vif* (Fig 3C and 3D). Interestingly, two human A3s (hA3B and hA3H hapII) showed strong inhibition of SIVcpz regardless of Vif expression (Fig 3C and 3D). While hA3H is expressed in primary CD4^+^ lymphocytes and has the ability to inhibit HIV-1 [26,35], hA3B is not found in HIV target cells [35]. Together our data indicate that hA3H hapII may block SIVcpz cross-species transmission to humans.

**Fig 4:**
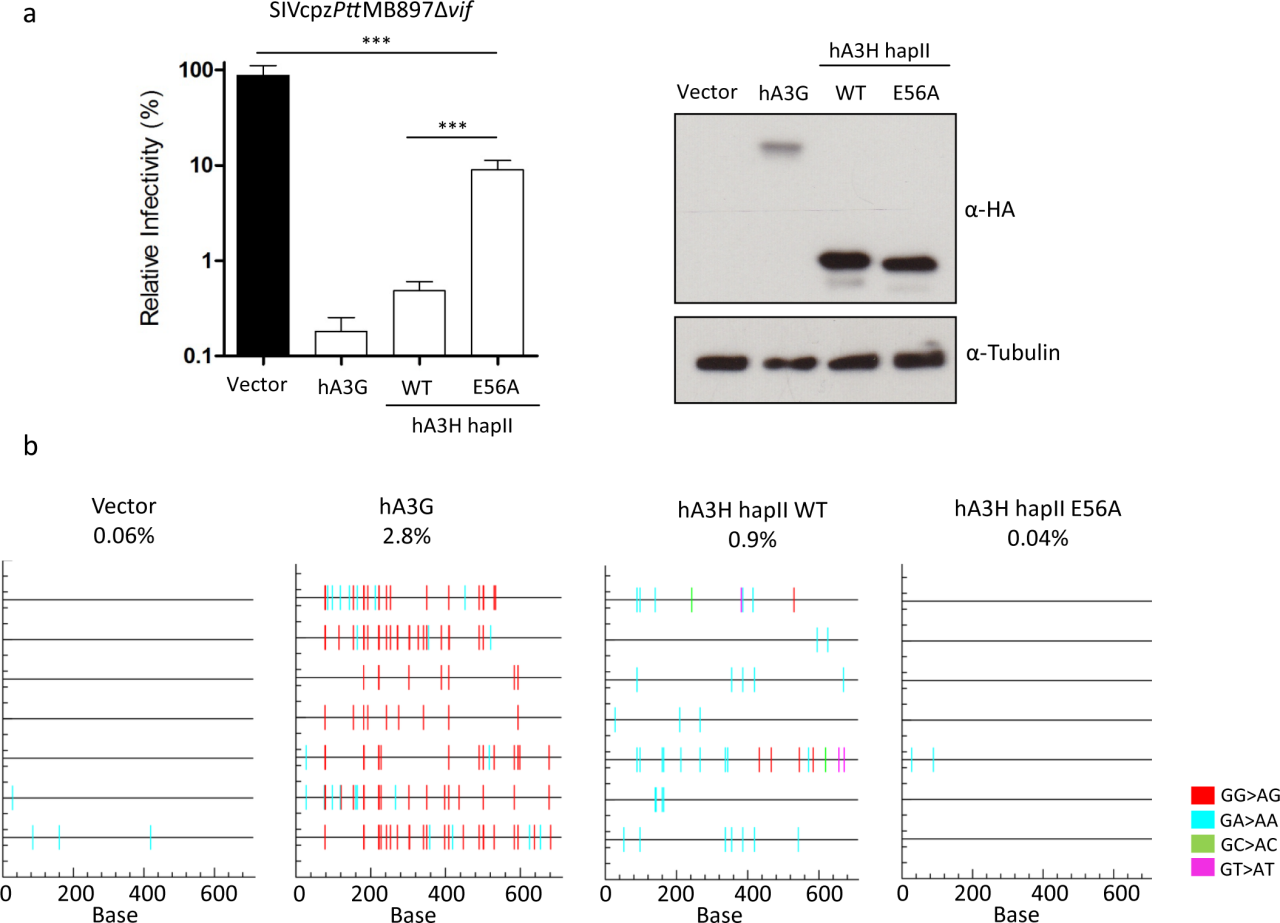
SIVcpz is inhibited by hA3H hapII by both deaminase dependent and independent mechanisms. (**a**) SIVcpz*Ptt*MB897Δ*vif* reporter constructs were co-transfected with hA3G, hA3H hapII or hA3H hapII E56A expression plasmids, PTR600 empty vector was used as control (vector). Two days post-transfection, normalized amounts of viruses were used to infect 293T cells. The nanoluciferase (relative light units-RLU) was measured two days post-infection. The expression of A3s was detected by anti-HA antibody, tubulin served as loading control. (**b**) The viral supernatants from (a) were treated with DNase I (20 units for 1 ml viral supernatant) and used to infect 293T cells. 12 h post-infection, the cellular DNA was isolated. A 700-bp fragment of SIVcpz was amplified by PCR, cloned and sequenced.

To characterize the interaction between SIVcpz and A3H in more detail, the incorporation of cpzA3H and hA3H hapII into SIVcpz viral particles was analyzed by immunoblotting. In the absence of Vif, both cpzA3H and hA3H hapII were encapsidated into SIVcpz*Ptt*MB897 and SIVcpz*Pts*TAN1 (S2D Fig). Vif from both SIVcpz strains reduced the cpzA3H protein level in the cell lysate by depletion and decreased the cpzA3H incorporation into viral particles (S2D Fig). In agreement with the infectivity data of SIVcpz with cpzA3H (Fig 2C and 2D), SIVcpz*Ptt* Vif was more active against cpzA3H than SIVcpz*Pts* Vif [40]. However, the steady-state expression and particle encapsidation of hA3H hapII did not change in the presence of SIVcpz Vif, which corresponds with hA3H’s hapII antiviral activity against wild-type SIVcpz (Fig 3C and 3D and S2D Fig). Furthermore, we investigated whether the cytidine deaminase activity is required for hA3H hapII inhibiting SIVcpz. We introduced the E56A mutation in the cytidine deaminase domain of hA3H hapII, which was previously reported to completely abolish the protein’s deaminase activity [42]. The E56A mutant of A3H lost significantly anti-viral activity compared with wild-type hA3H hapII, but remained a 10-fold inhibitory activity against SIVcpzΔ*vif* (Fig 4A). Next we analyzed the presence of G-to-A mutations indicative of A3 deamination in the viral genome by amplifying a 700-bp fragment of the viral genome 12 h post-infection. Viruses prepared without co-expression of A3 showed no detectable G-to-A mutations. However, in the presence of hA3G, we found a hypermutation rate of around 2.8% in the SIVcpz genome (Fig 4B). Viruses made in the presence of hA3H hapII contained a mutation rate of around 0.9%, while hA3H hapII E56A did not edit the SIVcpz genome (Fig 4B). The sequence plots, confirmed that hA3G preferred GG motif (mutated G is underlined, which is CC in the deaminated minus strand), while hA3H hapII mutated a GA motif (TC in the minus strand) predominantly (Fig 4B), which is consistent to previous studies [42–44]. Taken together, these data indicate that hA3H hapII inhibits SIVcpz by both deaminase dependent and independent mechanisms.

**Fig 5:**
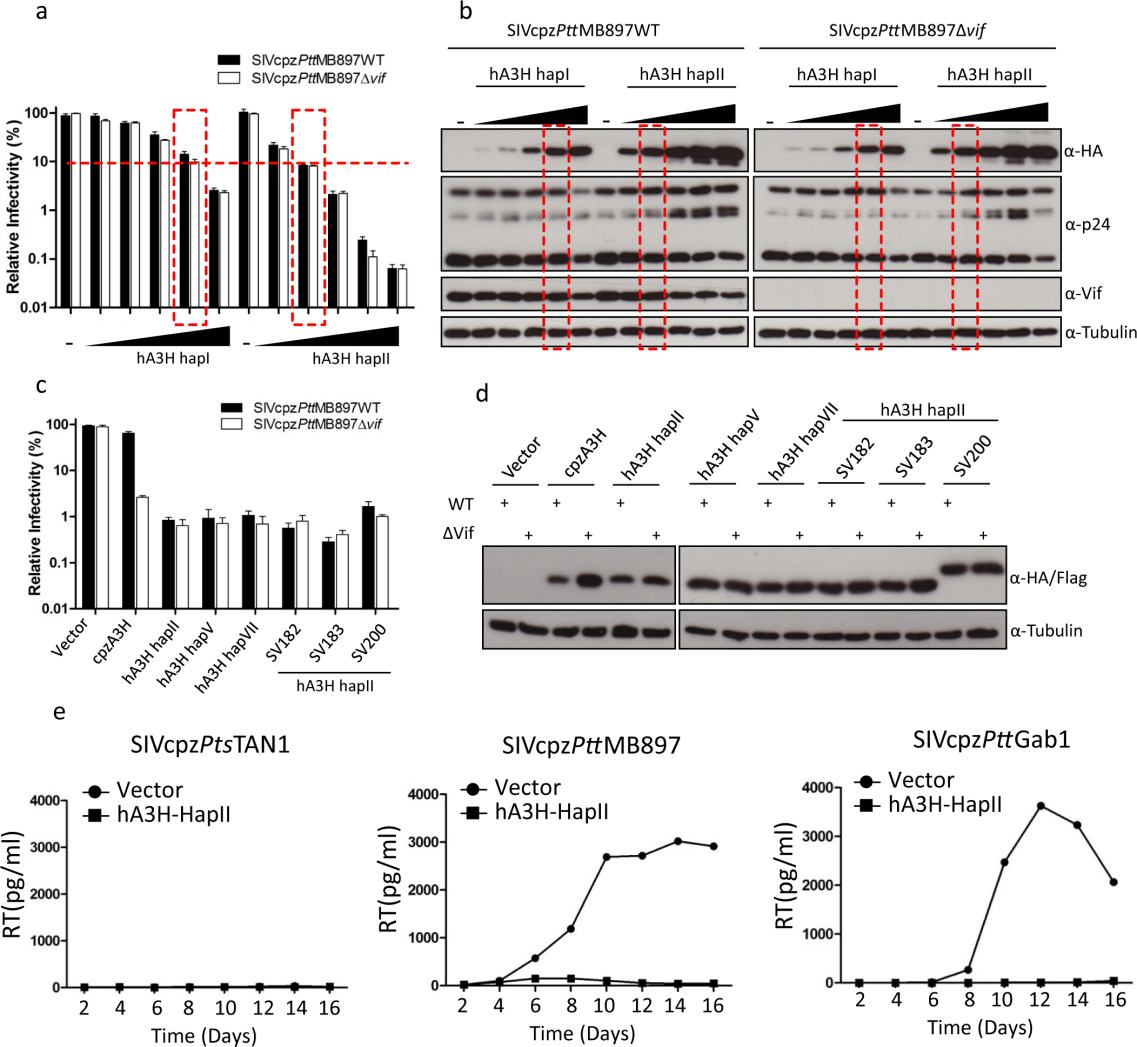
Inhibition of SIVcpz by hA3H haplotypes with stable protein expression. (**a**) SIVcpz*Ptt*MB897WT or SIVcpz*Ptt*MB897Δ*vif* reporter constructs were co-transfected with increasing amounts of hA3H hapI or hapII expression plasmids (5, 10, 30, 100 or 200 ng), PTR600 empty vector was used to bring the total transfected plasmid DNA to 600 ng and also used as control (vector). Two days post-transfection, normalized amounts of viruses were used to infect 293T cells. The nanoluciferase (relative light units-RLU) was measured two days post-infection. (**b**) Cell lysates from (a) were used to detect the expression of A3s, SIVcpz capsid (p24), or SIVcpz Vif by anti-HA, anti-p24, or anti-Vif antibody, respectively. Tubulin served as a loading control. (**c**) SIVcpz*Ptt*MB897WT or SIVcpz*Ptt*MB897Δ*vif* reporter constructs were co-transfected but with expression plasmids for different A3H haplotypes and splice variants. (**d**) Cell lysates from (c) were used to detect the expression of A3s by anti-HA antibody. Tubulin served as a loading control. (**e**) SupT11-vetor-hCCR5 or SupT11-hA3H hapII-hCCR5 cells were infected with 1 ng RT activity of SIVcpz*Pts*TAN1, SIVcpz*Ptt*MB897 or SIVcpz*Ptt*Gab1, respectively, and culture supernatants were collected each second day and quantified by the RT assay.

To further characterize the level of anti-SIVcpz activity mediated by hA3H, different amounts (5–200 ng) of hA3H hapI or hA3H hapII expression plasmids were co-transfected with SIVcpz*Ptt*MB897 wild-type or Δ*vif* reporter constructs and the viral infectivities were determined. The results indicate that the anti-SIVcpz activity of hA3H hapII increased with the dose of transfected hA3H hapII plasmid regardless of Vif expression (Fig 5A and 5B). Even a low level of hA3H hapII (5 ng) displayed around 10-fold inhibition of SIVcpz*Ptt*MB897Δ*vif* and Vif was not able to overcome this restriction. We also found that hA3H hapI showed around 20-fold inhibition of SIVcpz*Ptt*MB897, when 200 ng hA3H hapI expression plasmid was transfected (Fig 5A). 100 ng hA3H hapI and 10 ng hA3H hapII plasmids displayed similar protein expression levels, and they showed similar strength of inhibition of SIVcpz*Ptt*MB897 (Red box in Fig 5A and 5B), which is consistent with previous studies [45,46]. These results indicate that the protein expression level of A3H is one of the key determinants for its antiviral activity.

Human A3H has seven haplotypes and several splice variants, and the A3H protein stability determines the antiviral activity [29–31]. In the absence of Vif, cpzA3H, hA3H hapII, hA3H hapV, hA3H hapVII, and four hA3H hapII splice variants (SV182, SV183, and SV200) strongly inhibited SIVcpz*Ptt*MB897 (Fig 5C). However, Vif only counteracted the restriction of cpzA3H and was inactive against all the tested hA3H variants (Fig 5C). Corresponding immunoblotting results of lysates of the transfected cells confirmed that SIVcpz*Ptt*MB897 Vif only reduced the protein level of cpzA3H and protein levels of the hA3Hs were not changed by Vif co-expression (Fig 5D).

To learn more about the strength of hA3H’s antiviral activity, the spreading replication of SIVcpz in human T cells (SupT11) that stably expressed hA3H hapII [47] was investigated. To facilitate replication of CCR5-tropic SIVcpz, we modified SupT11 cells to express human CCR5 (S3A and S3B Fig). The spreading replication was tested with full-length unmodified viruses (SIVcpz*Ptt*MB897, SIVcpz*Ptt*Gab1, and SIVcpz*Pts*TAN1). SIVcpz*Pts*TAN1 did not replicate in the SupT11-hA3H hapII and SupT11-vector cells, regardless of the input of virus (1 ng reverse transcriptase (RT) activity or 50 ng RT) for the initial infection (Fig 5E and S3C Fig). Both SIVcpz*Ptt*MB897 and SIVcpz*Ptt*Gab1 replicated efficiently in SupT11-vector cells, while no virus spreading was observed in SupT11-hA3H hapII cells (Fig 5E). These data indicate that hA3H hapII is a strong inhibitor of infection of SIVcpz in human T cells. We conclude, therefore, that stably expressed hA3H variants are Vif-resistant restriction factors of SIVcpz.

### Identification of A3H residues that are important for antagonism by SIVcpz Vif

**Fig 6:**
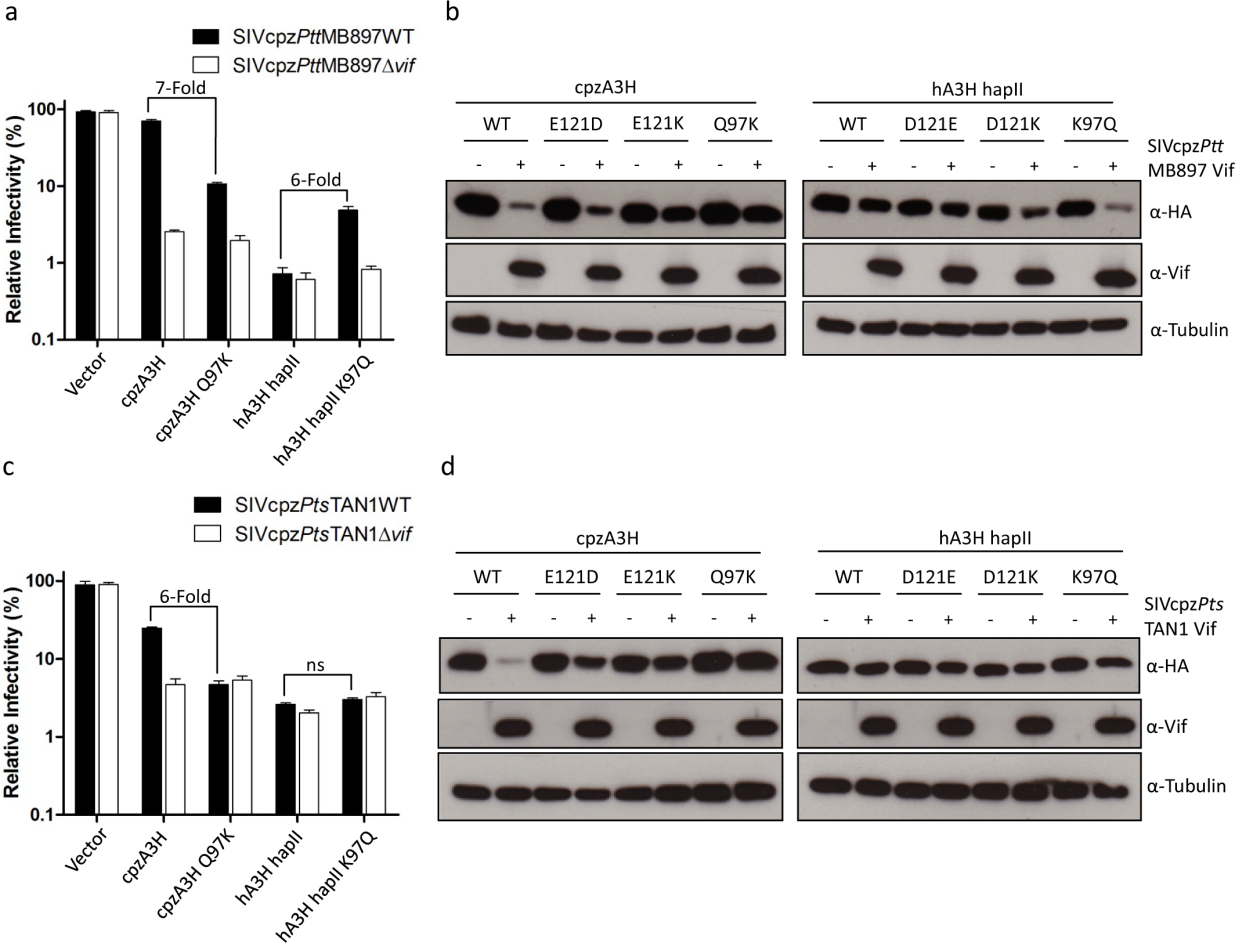
Residue 97 of A3H partially determines the sensitivity to SIVcpz Vifs. (**a, b**) SIVcpz*Ptt*MB897 or SIVcpz*Pts*TAN1 wild type or delta v*if* reporter viruses were produced in 293T cells in the presence of A3H wild type or mutant expression plasmids, as indicated. The viral infectivity was determined by measuring the nanoluciferase of 293T cells infected with normalized amounts of SIVcpz reporter viruses. (**c, d**) cpzA3H, hA3H wild type or mutants expression plasmids were co-transfected with SIVcpz (SIVcpz*Ptt*MB897 or SIVcpz*Pts*TAN1) Vif expression plasmids into 293T cells. The presence of A3H and Vif was detected by using anti-HA and anti-Vif antibodies, respectively. Tubulin served as a loading control.

Both cpzA3H and hA3H hapII displayed strong anti-SIVcpz activity, while they had different sensitivities to SIVcpz Vif counteraction (Figs 2C, 2D, 3C and 3D). One recent study demonstrated that residue 97 of cpzA3H and hA3H hapII determines the sensitivity to HIV-1 clone NL4-3 Vif [48]. Thus, we tested whether residue 97 would also be important for SIVcpz Vif inhibition of A3H. The Q97K and K97Q mutations were introduced into cpzA3H and hA3H hapII, respectively. The results showed that cpzA3H Q97K and hA3H hapII K97Q retained their anti-SIVcpz activity in the absence of Vif (Fig 6A and 6C). While SIVcpz*Ptt*MB897 Vif almost fully overcame the inhibition of wild-type cpzA3H, it only partially antagonized cpzA3H Q97K and, similarly, SIVcpz*Pts*TAN1 Vif did not counteract cpzA3H Q97K (Fig 6A and 6C). Additionally, hA3H hapII showed resistance to SIVcpz*Ptt*MB897 Vif, but this resistance was partially lost when the K97Q mutation was introduced (Fig 6A). In contrast to SIVcpz*Ptt*MB897 Vif, both wild-type hA3H hapII and its K97Q mutant showed resistance to SIVcpz*Pts*TAN1 Vif (Fig 6C). Furthermore, we analyzed the protein expression level of these A3H mutants in the presence of SIVcpz Vifs. hA3H E121K was included as a control mutant that could not be degraded by HIV-1 Vif [48,49]. SIVcpz*Ptt*MB897 Vif slightly reduced the protein level of cpzA3H Q97K compared to the no-Vif control, which is consistent with the infectivity data (Fig 6A and 6B). SIVcpz*Pts*TAN1 Vif did not affect the expression of cpzA3H Q97K (Fig 6D). hA3H hapII K97Q was depleted by co-expression of SIVcpz*Ptt*MB897 Vif, while the presence of SIVcpz*Pts*TAN1 Vif did not affect hA3H protein levels (Fig 6B and 6D). We conclude that Vifs from SIVcpz*Ptt*MB897 and SIVcpz*Pts*TAN1 have distinct interaction properties with hA3H hapII.

### The polymorphism of the cpzA3H gene in chimpanzees

**Fig 7:**
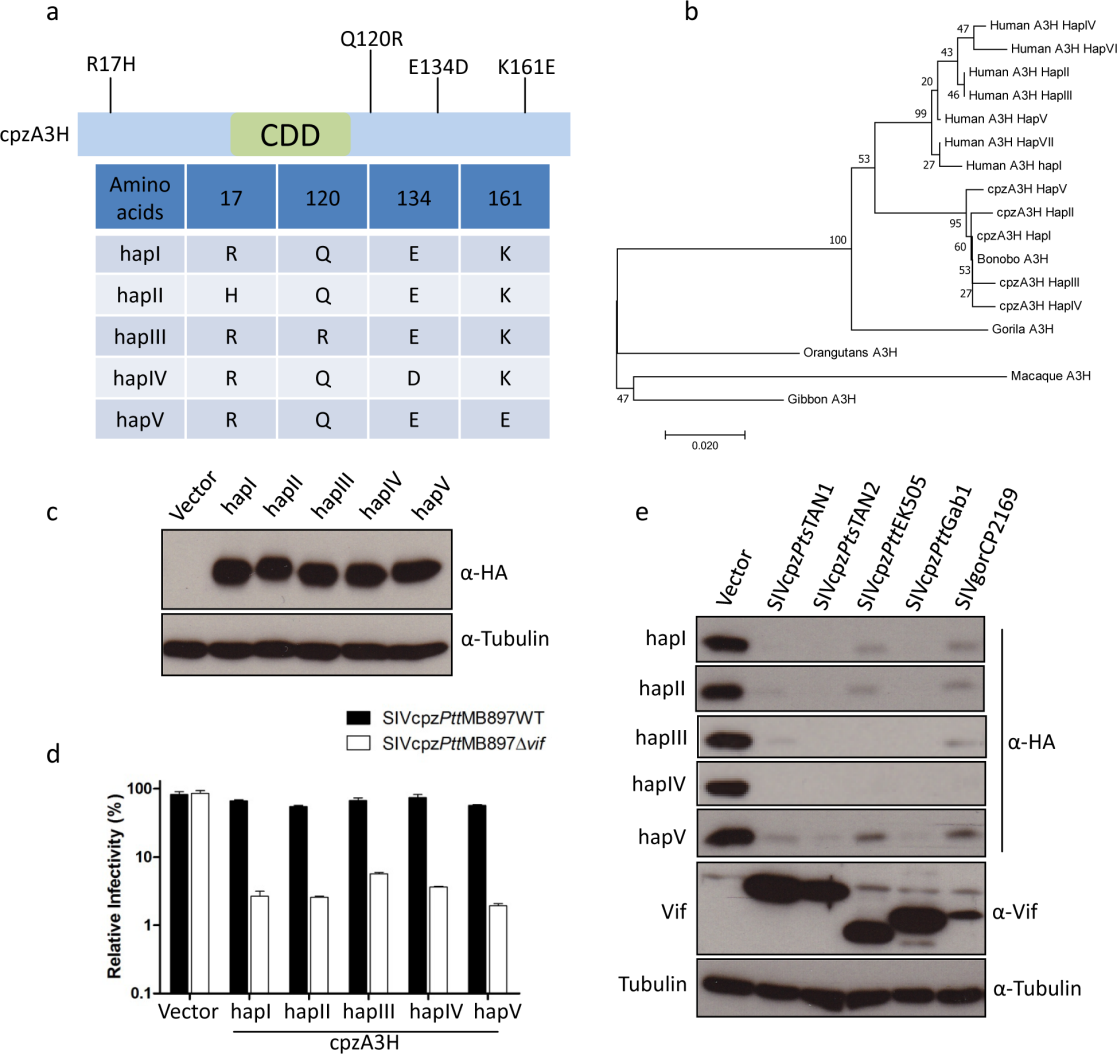
Similar anti-SIVcpz activity of cpzA3H haplotypes. (**a**) Four single nucleotide polymorphisms (SNPs) of cpzA3H were observed in 61 chimpanzees, see also Table 1. These four SNPs including reference cpzA3H were named haplotype I (hapI) to haplotype V (hapV). CDD, cytidine deaminase domain. (**b**) The polygenetic relationship of primate A3H proteins. 500 bootstrap replications were performed during calculation. The number on each node indicates the bootstrap support. (**c**) cpzA3H expression plasmids for different haplotypes were transfected into 293T cells. After two days, the expression of cpzA3H was detected by using anti-HA antibody, respectively. Tubulin served as a loading control. (**d**) SIVcpz*Ptt*MB897WT or SIVcpz*Ptt*MB897Δ*vif* reporter constructs were co-transfected with expression plasmids for cpzA3H haplotypes into 293T cells, pcDNA3.1(+) empty vector was used as control (vector). Two days post-transfection, normalized amounts of virus were used to infect 293T cells. The nanoluciferase (relative light units-RLU) was measured 2 days post-infection. (**e**) The expression plasmids of cpzA3H haplotypes were co-transfected with expression plasmids for Vifs (from different SIVcpz strains, as indicated) into 293T cells. The presence of A3H and Vif were detected by using anti-HA and anti-Vif antibodies, respectively. Tubulin served as a loading control.

To find out how diverse A3H is in chimpanzees, we analyzed the deep-sequencing reads from the recent Great Ape Genome Project [50]. We mapped reads to the hA3H region (hg19, chr22:39496284–39498576) and the exons of A3H were isolated. The coding regions of A3H from 61 chimpanzees (10 *Pan troglodytes ellioti*, *Pte*; 16 *Pan troglodytes schweinfurthii*, *Pts*; 22 *Pan troglodytes troglodytes*, *Ptt*; 13 *Pan troglodytes verus*, *Ptv*) were analyzed. We found four single-nucleotide polymorphisms (SNPs) of cpzA3H (nucleotide positions 50, 359, 402, and 481; Table 1, Fig 7A and S4 Fig). Two of them (SNP_50 and SNP_359) were only present in *Ptv* with an overall frequency of 6.5% and 9.8%, respectively. SNP_402 was only found in *Pts* with a frequency of 9%. However, SNP_481 was detected in *Pte*, *Pts*, and *Ptt* with a frequency of 8.2%, 19.6%, and 34.4%, respectively. The detailed SNP and zygosity information is described in Table 1. These four SNPs including the reference cpzA3H were named from haplotype I (hapI) to haplotype V (hapV) (Fig 7A). In addition, we performed a phylogenetic analysis of A3H from apes (rhesus macaque A3H was also included). The results showed that gibbon, rhesus macaque, and orangutan A3H were classified into one clade. Gorilla A3H formed a separate clade, and human and chimpanzee A3H were classified into two clades, respectively (Fig 7B). Bonobo A3H was classified into the clade of chimpanzee A3H.

**Table 1:** Genetic variants in the APOBEC3H gene in chimpanzee subspecies.

POS. NT^a^	REF	ALT	POS. AA^b^	REF	ALT	Summary of ALT^c^	HET^d^	HOM ALT^e^
50	G	A	17	R	H	4 Ptv	3	1
359	A	G	120	Q	R	6 Ptv	6	0
402	G	T	134	E	D	3 Pts	3	0
481	A	G	161	K	E	5 Pte, 12 Pts, 21 Ptt	18	20

a, position of nucleotide variant; REF, chimpanzee mRNA sequence from NCBI (NM_001142606.1); ALT, Variant.

b, position of corresponding amino acid in A3H protein.

c, summary of the individuals bearing the variant; Pts, *Pan troglodytes schweinfurthii*; Ptt, *P*. *t*. *troglodytes*; Pte, *P*. *t*. *ellioti*; Ptv, *P*. *t*.*verus*.

d, total number of individuals heterozygous.

e, total number of individuals homozygous for the variant.

The protein stability differs in human A3H haplotypes and it is one of the determinants of its antiviral activity [29–31]. Thus, the expression of five cpzA3H haplotypes in 293T cells was tested by immunoblotting. All cpzA3H haplotypes produce stable proteins and had similar expression levels (Fig 7C). Moreover, these five cpzA3H haplotypes displayed similar anti-SIVcpz activities and were all sensitive to Vifs from SIVcpz lineages (Fig 7D and 7E). These data indicate that the polymorphism of cpzA3H does not affect its protein stability or antiviral activity.

### Vifs from SIVcpz lineages fail to counteract human A3H haplotype II

**Fig 8:**
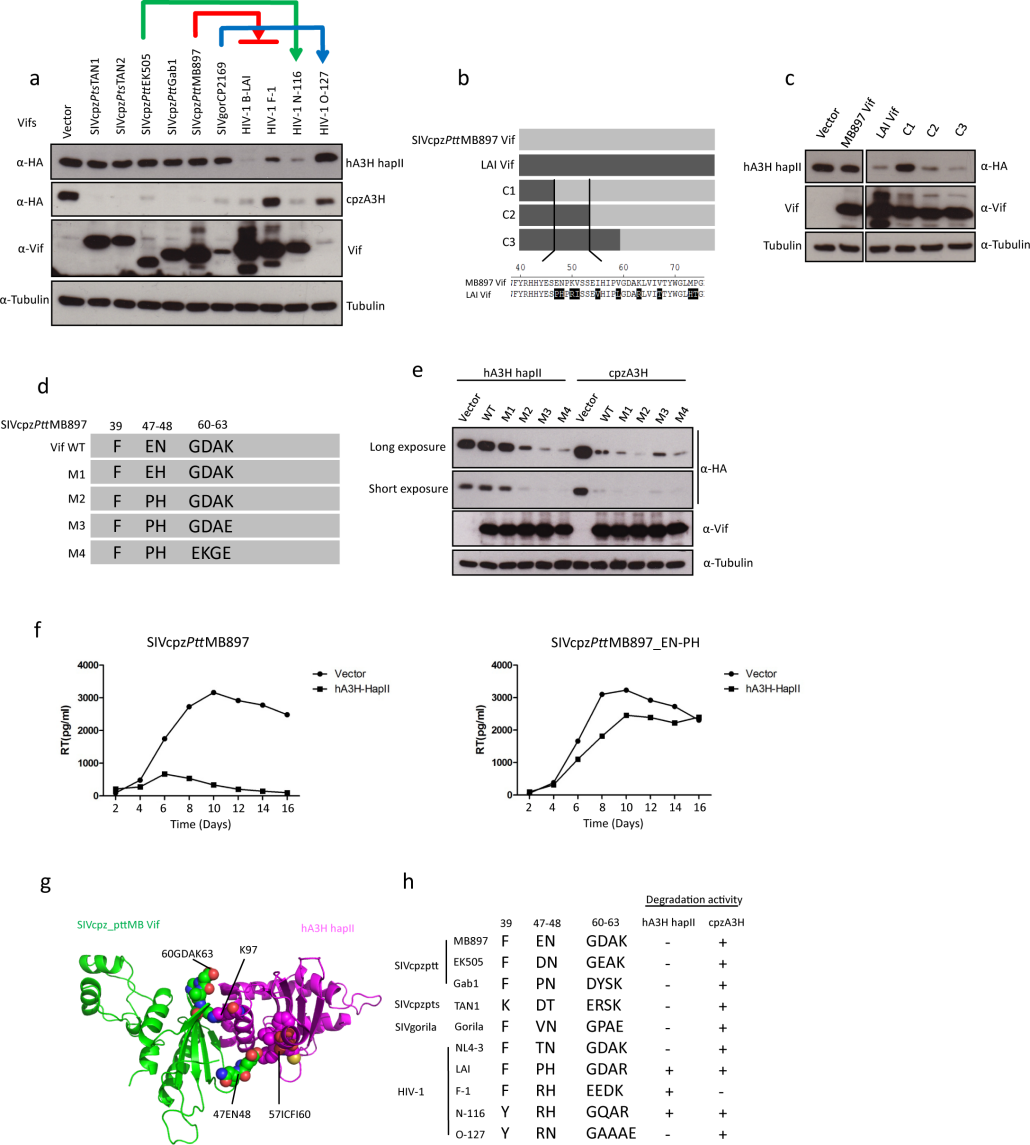
No counteraction of human A3H haplotype II by Vifs from different SIVcpz lineages. (**a**) cpzA3H or hA3H hapII wild type expression plasmids were co-transfected with expression plasmids for Vifs (different SIVcpz, SIVgor or HIV-1 strains, as indicated). The presence of A3H and Vif was detected by using anti-HA and anti-Vif antibodies, respectively. Tubulin served as a loading control. (**b**) The schematic structure of SIVcpz*Ptt*MB897 and HIV-1 LAI Vif chimeras. (**c**) hA3H hapII and Vif chimera expression plasmids were co-transfected into 293T cells. The presence of A3H and Vif was detected by using anti-HA and anti-Vif antibodies, respectively. Tubulin served as a loading control. (**d**) Schematic structure of SIVcpz*Ptt*MB897 Vif mutants. (**e**) cpzA3H or hA3H hapII wild type expression plasmids were co-transfected with expression plasmids for different SIVcpz*Ptt*MB897 Vif mutants. The presence of A3H and Vif was detected by using anti-HA and anti-Vif antibodies, respectively. Tubulin served as a loading control. (**f**) SIVcpz*Ptt*MB897 with the mutation of 47EN48-47PH48 in Vif and wild type SIVcpz (5 ng RT activity) were used to infect SupT11-vetor-hCCR5 or SupT11-hA3H hapII-hCCR5 cells, respectively. The viral supernatants were collected each second day and quantified by RT assay. (**g**) Superimposition of SIVcpz Vif-hA3H hapII model structure. 47EN48 and 60GDAK63 motifs are shown by sphere, and their contact with hA3H hapII is displayed. (**h**) Summary: Correlation between Vif from different SIVcpz and HIV-1 lineages and antagonism activity against cpzA3H and hA3H hapII. Identity of amino acids at important positions (39, 47–48 and 60–63) of Vif was shown. -, + represents low and high A3H antagonism.

There have been four independent transmissions from different SIVcpz/gor strains to the human population, which caused HIV-1 groups M, N, O, and P, respectively [11,14]. Thus, we tested the sensitivity of cpzA3H and hA3H hapII to Vifs from several SIVcpz/HIV-1 lineages. The immunoblots of co-expressing cells indicated that cpzA3H was depleted by all the tested SIVcpz Vifs, and it was also depleted by Vifs from HIV-1 B-LAI (M group), N-116, and O-127, but was not degraded by HIV-1 F-1 Vif (Fig 8A). hA3H hapII was resistant to depletion of all SIVcpz Vifs tested, including SIVgor Vif (Fig 8A). However, HIV-1 B-LAI, F-1, and N-116 Vifs induced the degradation of hA3H hapII (Fig 8A). Unexpectedly, HIV-1 O-127 Vif, which protein expression was not detectable was inactive against hA3H hapII (Fig 8A).

By testing chimeras of SIVcpz*Ptt*MB897 and HIV-1 LAI Vif, we identified that the Vif N-terminal region (residues 40–70) is essential for hA3H hapII depletion (Fig 8B and 8C). A previous study described the importance of HIV-1 Vif residues F39 and H48 for antagonism of hA3H hapII [33]. F39 is present in SIVcpz*Ptt*MB897 Vif, but at the 48 position, an asparagine (N) is found (Fig 8D). However, introducing an N48H mutation (construct M1) in SIVcpz*Ptt*MB897 Vif did not promote degradation of hA3H hapII (Fig 8E). However, the local area of residue 48 of SIVcpz*Ptt*MB897 Vif was important as an additional mutation revealed that changing residues 47EN48 to 47PH48 (construct M2) facilitated hA3H hapII depletion (Fig 8E). Furthermore, a replication-competent SIVcpz*Ptt*MB897_EN-PH with this substitution showed spreading replication in hA3H hapII-containing SupT11 cells (Fig 8F).

A previous study showed that HIV-1 Vif from a homozygous hA3H haplotype II patient had greater activity against hA3H hapII compared to other laboratory HIV-1 Vifs, which correlated with the presence of four amino acid substitutions (60GDAK63 to 60EKGE63) [32]. This substitution was introduced into SIVcpz*Ptt*MB897 Vif and led to enhanced hA3H hapII depletion (Fig 8E, Vif M2 compared to M3 and M4). Based on a recent HIV-1 Vif-hA3H hapII co-structure model [49], the co-structure of SIVcpz*Ptt*MB897 Vif-hA3H hapII was modeled. From the structure, we found that residues 47EN48 and 60GDAK63 of SIVcpz*Ptt*MB897 Vif were in close contact with hA3H hapII (Fig 8G). Both regions are diverse in Vifs from distinct SIVcpz and HIV-1 lineages (Fig 8H).

**Fig 9:**
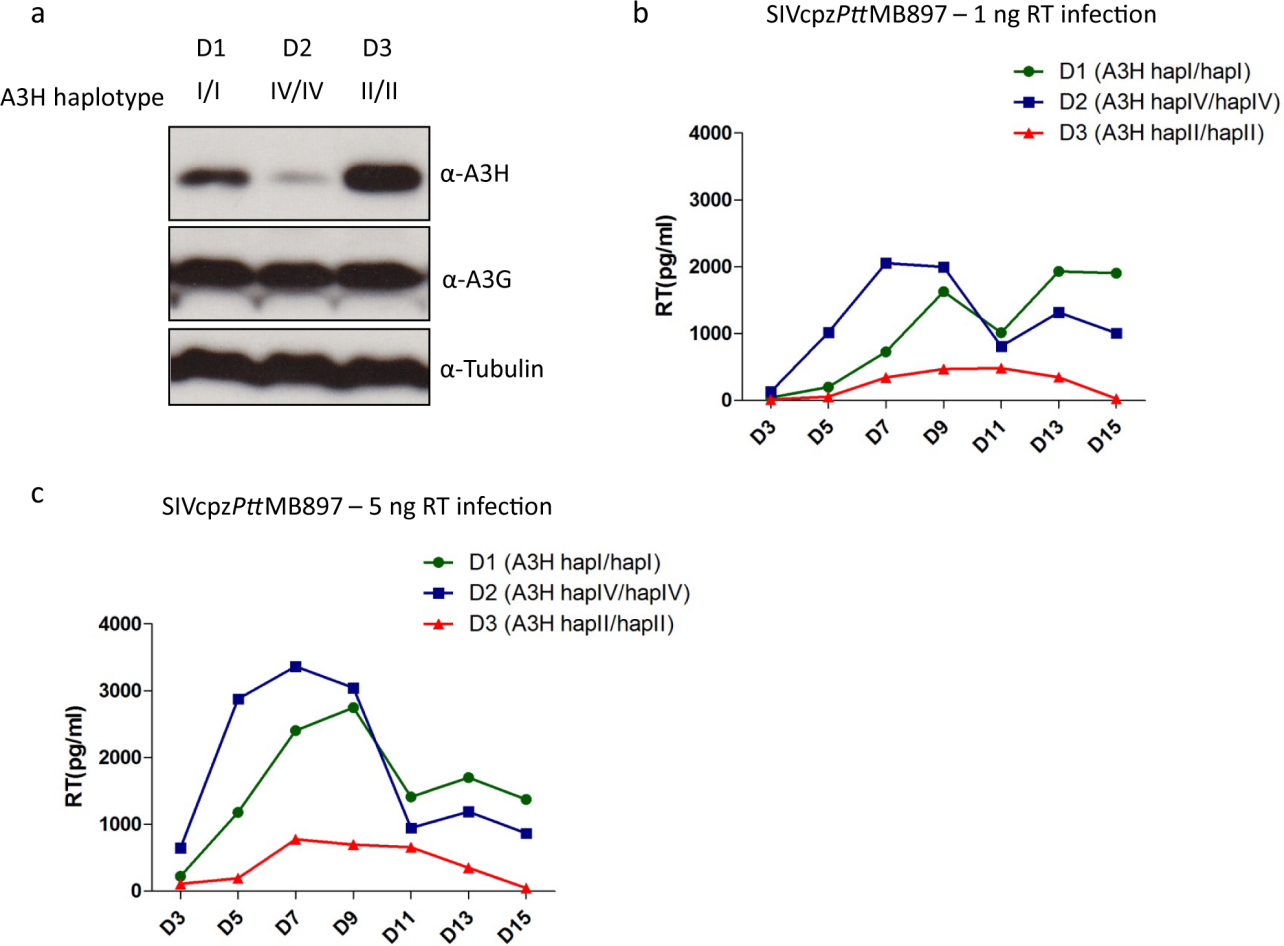
Stable hA3H inhibits SIVcpz replication in human PBMCs. (**a**) The genotypes of different donors were determined by sequencing the A3H mRNAs. Expression of A3H and A3G proteins in stimulated PBMCs were detected by immunoblots using anti-hA3H and anti-hA3G antibodies, respectively. Tubulin served as a loading control. (**b, c**) PBMCs from different donors were infected with 1 ng RT or 5 ng RT activity of SIVcpz*Ptt*MB897, respectively, and culture supernatants were collected each 2–3 day and analyzed for the RT activity.

**Fig 10:**
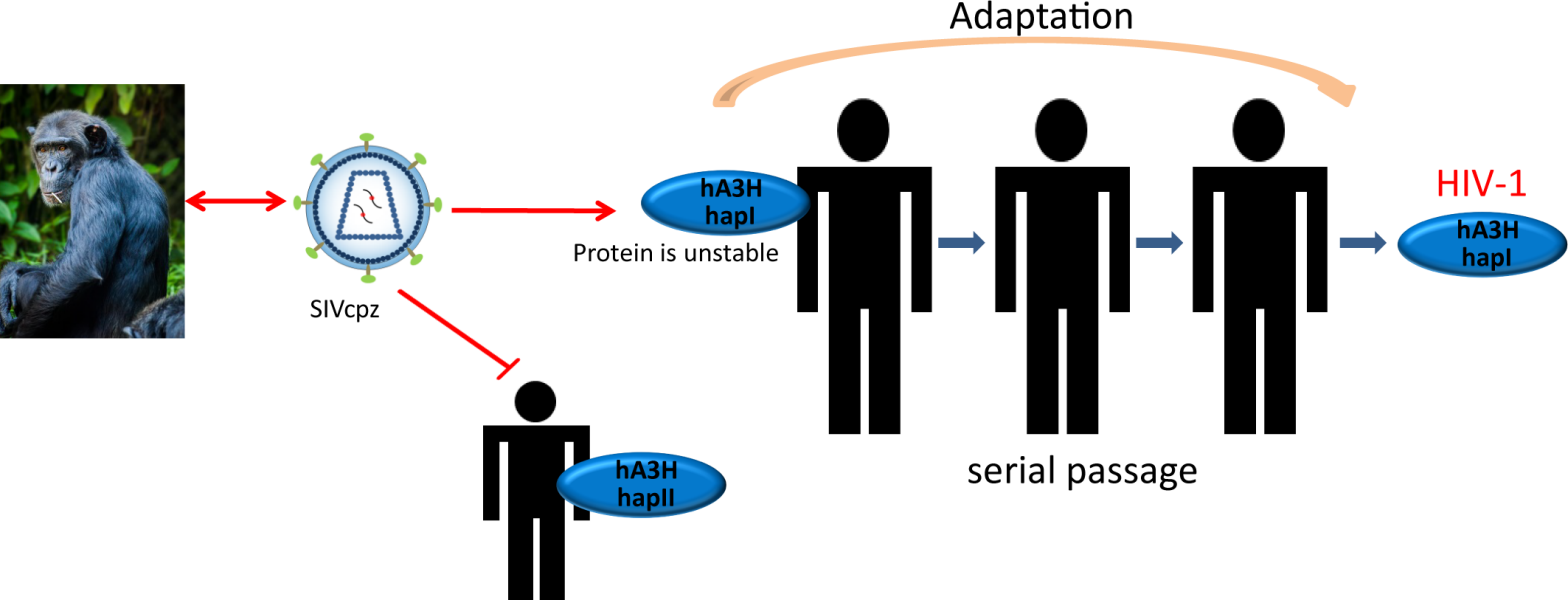
The model of SIVcpz cross species transmission to human. The model predicts that cross-species transmission of SIVcpz is blocked by hA3H hapII (or other stably expressed variants), but this transmission is easier obtained in humans with unstable A3H haplotypes.

Next, we tested the replication of SIVcpz in human PBMCs from donors with different hA3H genotypes. We identified three donors who were homozygous for A3H hapI, hapIV and hapII, respectively. The protein expression of A3H in stimulated PBMCs was detected by immunoblotting, demonstrating highest protein levels in the PBMCs of hapII, moderate levels in PBMCs of hapI and weak levels in cells of hapIV (Fig 9A). However, the A3G expression in these PBMC was identical (Fig 9A). The viral replication experiments indicated that SIVcpz*Ptt*MB897 replicated fastest in PBMCs from the donor with haplotype IV, and moderately in PBMCs from the donor with haplotype I (Fig 9B and 9C). However, the replication of SIVcpz*Ptt*MB897 was inhibited in PBMCs from donor with haplotype II (Fig 9B and 9C). In summary, we speculate that stable hA3H forms a barrier for zoonotic transmission of SIVcpz to humans and Vif adaptation to stable hA3H would be needed for high-level infection of humans with this haplotype (Fig 10).

## Discussion

SIVcpz originated from the cross-species transmission and recombination of three different SIVs [5,6]. After lentiviral transmission to a new host that differs in one or many A3 proteins, Vif adaptation is expected at the interface of both proteins [25,51]. In our study, all tested SIVcpz Vifs had the ability to counteract cpzA3Hs (Figs 2 and 7). Lucie Etienne *et al*. found that SIVrcm Vif acts like SIVcpz Vifs and can neutralize cpzA3H, while SIVmus Vif could not antagonize the restriction of cpzA3H [40]. Overcoming the restriction of cpzA3H may be one explanation for SIVcpz selectively acquiring the 5’ region (including *vif*) from SIVrcm during recombination, and acquiring the 3’ region (including *vpu*, *env*, and *nef*) from SIVgsn/SIVmus/SIVmon may have facilitated the counteraction of other restriction factors, such as Tetherin or Serinc3/5 [52–54]. Here, we also found that cpzA3D, F, and G were resistant to SIVagm Vif and similarly, cpzA3D was resistant to SIVmac Vif, confirming a previous report [40]. This observation indicates that cpzA3D and cpzA3G can protect chimpanzees from infection with SIVs of rhesus macaques and African green monkeys. On the other hand, SIVcpz Vifs could counteract all the tested cpzA3s. However, cpzA3F showed a moderate level of resistance to degradation induced by SIVcpz Vif (Fig 2C and 2D), possible suggesting that cpzA3F may provide some repression of SIVcpz infection. Human and chimpanzee A3D, F, and G display a similar sensitivity to SIVcpz Vif, indicating that the inhibitory activity against cpzA3s by SIVcpz may be a prerequisite for the cross-species transmission of SIVcpz to the human population.

Here, we found that hA3C and hA3H hapI display a strong restriction against SIVmacΔ*vif* and SIVagmΔ*vif*; however, no antiviral activity was observed against SIVcpzΔ*vif* (Fig 3) or HIV-1Δ*vif* [26,41,55]. These data suggest that the viral sensitivity to hA3C and hA3H hapI was lost in the evolution of SIV lineages and not during the evolution of HIV-1. We cannot determine whether this happens during the creation of SIVcpz due to the lack of information regarding the antiviral activity of hA3C and hA3H hapI against SIVrcm/SIVgsn/SIVmus/SIVmon. We speculate that some SIVs similar to HIV-1 have the ability to escape hA3C and hA3H hapI restriction by a Vif-independent mechanism [55].

cpzA3H appears to be much less polymorphic than hA3H. However, A3F and A3G in chimpanzee are more diverse than the human orthologs [40]. Although our chimpanzee sample number was limited (61 chimpanzees), the results suggest that cpzA3H is relatively conserved among chimpanzees. Residues 15 and 105 of hA3H determine the protein stability and anti-viral activity [28]. However, no variability was identified at these two positions in cpzA3H, which is in agreement with the comparable protein stability and anti-viral activity of the currently recognized five cpzA3H haplotypes (Fig 7). Vifs of different SIVcpz isolates degrade all haplotypes of cpzA3H indicating that cpzA3H is not a restriction factor for inter-subspecies transmission of SIVcpz. Compared to cpzA3H, human A3H is more diverse and includes seven haplotypes and several splice variants [28,30,31]. In our study, the stably expressed hA3H haplotypes were identified as Vif-resistant inhibitors against SIVcpz, indicating that these active hA3Hs are strong barriers to prevent SIVcpz infection of humans. After the zoonotic transmission of SIVcpz to humans expressing unstable A3H haplotypes, the very early human-to-human transmission was likely to be severely affected by humans expressing the A3H haplotypes with a stable protein (Figs 9 and 10). A possible mutation that would enhance SIVcpz Vif adaptation was investigated by replacing residues 47EN48 of SIVcpz*Ptt*MB897 Vif with 47PH48 (Fig 8). It is possible—but unlikely—that there are currently not identified viruses circulating in chimpanzees with *vif* genes encoding 47PH48 residues enhancing SIVcpz cross-species transmission to humans. In fact, the 47PH48 motif is also found in HIV-1 patients who harbor hA3H hapII [32,34]. The frequency of active hA3H varies significantly between populations, with the highest frequency in Africans (around 50% harbor stable A3H) [28,30]. This observation may be the result of a selective sweep caused by exposure to a retrovirus such as SIV or HTLV or other A3H-sensitive pathogens [56,57]. Several previous studies described a positive and balancing selection of human and chimpanzee MHC loci, caused by HIV-1/SIVcpz infections [56,58–60].

In addition to hA3H hapII, human tetherin is also a strong barrier against SIVcpz transmission to humans. SIVcpz Nef recognizes the cytoplasmic domain of chimpanzee tetherin and inhibits its restriction, but it cannot overcome the restriction of human tetherin due to a deletion in this domain [54]. However, the virus adapts to this restriction by regaining Vpu-mediated inhibition of tetherin after transmission of SIVcpz to humans [54]. In fact, other unknown restriction factors may exist to control the cross-transmission of SIVcpz to humans. For example, a recent study found that introducing a M30R/K mutation in the Gag matrix could enhance SIVcpz replication fitness in human tonsil explant cultures [38].

Overall, our study suggests that the stable active human A3Hs can protect humans against the spillover of SIVcpz, and SIVcpz cross-species transmission to humans may have started in those that harbored unstable A3H proteins.

## Methods

### Plasmids

Chimpanzee APOBEC3 (A3) expression plasmids (A3D, A3F, A3G and A3H) were provided by Michael Emerman [40], chimpanzee A3C plasmid was described recently [61]. Human A3s (A3A-A3H) were expressed by PTR600 vector with a carboxyl-terminal triple hemaggutinin (HA) tag [33]. Human A3H haplotype V, VII, splice variants and E56A of haplotype II expression plasmids with a carboxyl-terminal flag tag were provided by Viviana Simon [31]. Human A3H haplotype II with an N terminal HA tag was re-cloned into PTR600 vector by using standard PCR. All human and chimpanzee A3H mutants were generated by site direct mutagenesis and confirmed by sequencing. The MLV packaging construct pHIT60 was kindly provided by Jonathan Stoye, which encodes the *gag-pol* of MoMLV [62]. The Plasmid of pBABE.CCR5 that encodes human CCR5 was obtained from NIH AIDSREPOSITORY [63]. SIVmac-Luc (R-E-), SIVmac-Luc (R-E-)Δ*vif* and SIVagm-Luc (R-E-) and SIVagm-Luc (R-E-)Δ*vif* were provided by N. R. Landau [64]. The replication competent SIVcpz*Ptt* clones MB897, EK505, Gab1 were kindly provided by Frank Kirchhoff [38,65]. SIVcpz*Pts* clones TAN1.910 and TAN2.69 and SIVgor clone CP2139 were obtained from NIH AIDSREPOSITORY [10,66]. To generate the Nanoluciferase reporter virus of SIVcpz*Ptt*MB897, the *nef* gene was replaced (the first 7 amino acids of Nef remained) by nanoluciferase gene by overlapping PCR using *NheI* and *XhoI* restriction sites. Additionally, two stop codons were inserted amino-terminal of Vif (amino acid position 40 and 44) by overlapping extension PCR using *PshAI* and *NheI* restriction sites. The same method was performed to create nanoluciferase reporter virus of SIVcpz*Pts*TAN1, and the restriction sites are shown in S1 Fig. Simply, the *nef* gene was replaced (the first 7 amino acids of Nef remained) by nanoluciferase gene by overlapping PCR using *AclIII* and *XbaI* restriction sites. Additionally, two stop codons were inserted at the amino-terminal of Vif (amino acid position 40 and 44) by overlapping extension PCR using *PshAI* and *AclI* restriction sites. All constructs were verified by sequencing analysis. To generate the SIV Vif expression plasmids, Vif fragments from the following molecular clones: SIVcpz*Ptt* EK505 (DQ373065), Gab1 (X52154), MB897 (EF535994) and SIVcpz*Pts* TAN1 (AF447763), TAN2 (DQ374657) and SIVgor CP2139 (FJ424866) were amplified and inserted into pCRV1 by *EcoRI* and *NotI*. Vif expression plasmids of HIV-1 LAI, F-1, N-116 and O-127 were provided by Viviana Simon [33,61]. All SIVcpz*Ptt*MB897 Vif mutants were generated by overlapping PCR and cloned into pCRV1 without any tag, verified by sequencing.

### Cells and single-round infection assay

HEK293T (293T, ATCC CRL-3216) cells were maintained in Dulbecco’s high-glucose modified Eagle’s medium (DMEM, Biochrom, Berlin, Germany) supplemented with 10% fetal bovine serum (FBS), 2 mM L-glutamine, penicillin (100 U/ml), and streptomycin (100 μg/ml). SupT11 cells containing empty control and hA3H hapII were kindly provided by Reuben S. Harris and cultured in RPMI supplemented with 10% fetal bovine serum (FBS), 2 mM L-glutamine, penicillin (100 U/ml), and streptomycin (100 μg/ml) [47]. SupT11 cells with expression of hCCR5 were generated by MLV transduction. Simply, 1x10^6^ SupT11 cells were transduced by MLV vector (produced by transfecting pBABE.CCR5, pHIT60 and VSV-G expression plasmid into 293T cells). 3 days after transduction, the SupT11 cells were selected for 3 weeks by using 1 μg/ml puromycin. For producing the single round infection of SIV reporter virus, 3×10^5^ 293T cells in 24-well plates were co-transfected with 300 ng SIVmac-Luc (R-E-), or SIVagm-Luc, or SIVcpz*Ptt*MB897-NLuc, or SIVcpz*Pts*TAN1-NLuc; or the corresponding delta Vif versions, 30 ng human A3s or 200 ng chimpanzee A3s expression plasmids and 50 ng VSV-G (pMD.G), and pcDNA3.1(+) (Thermo Fisher Scientific) was used instead of A3 expression plasmids. Human A3s were expressed in plasmid PTR600, while chimpanzee A3s were expressed in plasmid pcDNA3.1(+). 30 ng of PTR600-human A3s constructs had comparable expression levels with 200 ng pcDNA3.1(+)-chimpanzee A3s plasmids. Transfections were performed by using Lipofectamine LTX (Thermo Fisher Scientific) according to manufacturer’s instruction. The viral supernatants were collected 48 h post transfection. The reverse transcriptase (RT) activities of viruses were quantified by using the Cavidi HS lenti RT kit (Cavidi Tech, Uppsala, Sweden). For SIVmac and SIVagm infections, 5×10^4^ 293T cells were seeded in 96-well plates one day before transduction, and 50 pg RT of viruses were used for infection. After 48 h, firefly luciferase activity was measured with Steady-Glo Luciferase system (Promega) according to the manufacturer’s instructions on a MicroLumat Plus luminometer (Berthold Detection Systems, Pforzheim, Germany). For SIVcpz-NLuc, we observed high nanoluciferase enzyme activity in cell supernatant of transfected cells. 293T cells in 96-well plates were infected with 20 pg of SIVcpz*Ptt*MB897-NLuc or SIVcpz*Pts*TAN1-NLuc. To eliminate the effect of contaminating nanoluciferase in the supernatant of virus producer cells, we changed the medium 8 h post infection. 48 h after transduction, the cells were carefully washed by PBS once, and the nanoluciferase activity was measured with Nano-Glo Luciferase system (Promega) on a MicroLumat Plus luminometer (Berthold Detection Systems). Each sample was analyzed in triplicates; the error bar of each triplicate was shown. Infections in which the VSV-G glycoprotein was omitted served as control for nanoluciferase enzyme background enzyme activity.

### Detection of A3-mediated editing of SIVcpz transcripts

1 x 10^6^ 293T cells were infected with DNase I (Thermo Fisher, Germany) treated SIVcpzΔ*Vif*-Nluc produced in 293T cells together with hA3G, hA3H hapII, hA3H hapII E56A or pcDNA3.1(+). At 12 h post-infection, cells were washed with PBS, and DNA was isolated using a DNasy DNA insolation kit (Qiagen, Germany). A 700-bp fragment of the SIVcpz-Nluc (200-bp C terminal of *env* plus 500-bp nanoluciferase gene) was amplified using Dream*Taq* DNA polymerase (Thermo Fisher, Germany) with primers: 5’-attctccagtattggggacaagag-3’ and 5’-ttacgccagaatgcgttcgcac-3’. The PCR parameters were: 95°C for 5 min; 30 cycles with 88°C for 30 s, 57°C for 30 s, 72°C for 1min; 10 min at 72°C. PCR products were cloned using CloneJET PCR cloning kit (Thermo Scientific). Seven to ten clones were sequenced for each sample. A3 induced hypermutations were analyzed with the Hypermute online tool (http://www.hiv.lanl.gov/content/sequence/HYPERMUT/hypermut.html). The overall mutation rate was calculated by using the total number of G-A mutations divided by the total analyzed nucleotides.

### Flow cytometry

To analyze CD4 and CCR5 expression level of SupT11 cell lines, 5×10^5^ cells were stained by α-hCD4 PE mouse IgG1_k_ (Dako, Hamburg, Germany) and α-hCCR5 FITC (BD Bioscience, Heidelberg, Germany) separately according to the manufacturer’s instruction. The mouse IgG1/RPE isopeptidase was used as negative antibody control for CD4 staining. The measurement was carried out by BD FACSanto (BD Bioscience). Data analysis was done with the Software FlowJo version 7.6 (FlowJo, Ashland, USA).

### Ethics statement

Buffy-coats obtained from anonymous blood donors were obtained from University Hospital Düsseldorf blood bank. Whole blood was obtained from healthy and de-identified African donors that signed an informed consent. The research has been approved by the Ethics Committee of the Medical Faculty of the Heinrich-Heine-University Düsseldorf (Reference No 4767R - 2014072657) and performed according to the principles expressed in the Declaration of Helsinki.

### Determining of A3H haplotype expressed

Cellular RNA from PHA stimulated human PBMCs was isolated by using QIAGEN RNA extraction kit (Qiagen). 1 μg of total cellular RNA was used for reverse transcription with the RevertAid H Minus First Strand cDNA synthesis kit (Thermo Scientific). Human A3H cDNA was amplified with Q5 High-Fidelity DNA Polymerase (New England Biolabs) using primers: 5’-atggctctgttaacagccgaaacattcc-3’ and 5’-ggactgctttatcctgtcaagccgtcgc-3’. PCR products were cloned using CloneJET PCR cloning kit (Thermo Scientific). Six to ten clones were sequenced for each donor.

### SIVcpz replication on SupT11 cell lines

To produce SIVcpz, 1×10^6^ 293T cells in 6-well plate were transfected with 2 μg SIVcpz molecular clone plasmids (SIVcpz*Pts*Tan1, SIVcpz*Ptt*MB897 and SIVcpz*Ptt*Gab1). 2 days after transfection, the viral supernatants were collected and centrifuged at 12,000 rpm for 10 min to remove cell debris. Then the viral supernatants were concentrated through 20% sucrose cushion at 14,800 rpm 4 h, followed by resuspension in RPMI. The viral stock was quantified by using the Cavidi HS lenti RT kit (Cavidi Tech, Uppsala, Sweden). 5×10^5^ cells of each SupT11 cell lines (SupT11-vector-hCCR5 and SupT11-hA3H hapII-hCCR5) were infected with 1 ng or 5 ng RT activity of SIVcpz in a 24-well plate (in 500 μl) and cells were washed with PBS 1 day post-infection. Each second day, 200 μl supernatant was collected, clarified, and stored at ­80°C, and cultures were supplemented with fresh media. The replications were performed in two independent experiments, and each infection was performed in duplicates.

### SIVcpz replication on PBMCs

3 x 10^5^ PHA stimulated PBMC from three donors were infected overnight with SIVcpz*Ptt*MB897 representing either 1 ng RT activity or 5 ng RT activity in the presence of 30 U/ml Interleukin-2 (IL-2) in 96-well round-bottom plates (total volume 200 μl). After infection, cells were washed three times and maintained in complete RPMI with 30 U/ml of IL-2 for 15 days. 100 μl culture supernatant was collected every 2–3 days, and cultures were supplemented with fresh media. The RT activities of viruses in culture supernatant were quantified by using the Cavidi HS lenti RT kit (Cavidi Tech, Uppsala, Sweden).

### APOBEC3 degradation assay

A total of 3×10^5^ 293T cells in 24-well plates were co-transfected with 200 ng chimpanzee A3H expression plasmid or 50 ng hA3H haplotype II in PTR600 expression vector and 300 ng pCRV1 Vif expression plasmids, pcDNA3.1(+) (Thermo Fisher Scientific) was used to fill up the total transfected plasmid DNA to 500 ng. Transfections were performed by using Lipofectamine LTX (Thermo Fisher Scientific). 48 h post transfection, cells were lysed and clarified by 14,000 rpm/30 mins centrifugation. The expression of A3H and Vif were analyzed by immunoblots.

### Immunoblot analysis

Transfected 293T cells were lysed in radioimmunoprecipitation assay (RIPA) buffer (25 mM Tris-HCl [pH 8.0], 137 mM NaCl, 1% NP-40, 1% glycerol, 0.5% sodium deoxycholate, 0.1% sodium dodecyl sulfate [SDS], 2 mM EDTA, and protease inhibitor cocktail set III [Calbiochem, Darmstadt, Germany]). To pellet virions, culture supernatant were centrifuged at 12,000 rpm for 10 min followed by centrifugation through 20% sucrose cushion at 14,500 rpm 4 h and resuspended in RIPA buffer, boiled at 95⁰C for 5 min with Roti load reducing loading buffer (Carl Roth, Karlsruhe, Germany) and resolved on a SDS-PAGE gel. The expression of A3s and SIV/HIV Vifs were detected by mouse anti-hemagglutinin (anti-HA) antibody (1:7,500 dilution, MMS-101P; Covance, Münster, Germany), rabbit anti-HA antibody (1:1,000 dilution, C29F4, cat. 3724, Cell Signaling, USA) and rabbit anti-Vif polyclonal antibody (1:1,000 dilution, NIH AIDSREAGENTS, cat. 2221) [67]; tubulin and SIVcpz*Ptt*MB897 capsid protein was detected using mouse anti-α-tubulin antibody (1:4,000 dilution, clone B5-1-2; Sigma-Aldrich, Taufkirchen, Germany) and mouse anti-capsid p24/p27 MAb AG3.0 (1:50 dilution) separately [68], followed by horseradish peroxidase-conjugated rabbit anti-mouse or donkey anti-rabbit antibodies (α-mouse or rabbit-IgG-HRP; GE Healthcare, Munich, Germany), and developed with ECL chemiluminescence reagents (GE Healthcare). The expression of A3H in SupT11 cell lines was detected by using anti-hA3H (1:1,000) antibody [35] followed by horseradish peroxidase-conjugated rabbit anti-mouse and developed with ECL chemiluminescence reagents. 8 x 10^6^ human PBMCs from three donors were lysed in 100 μl RIPA buffer with protease inhibitor cocktail set III [Calbiochem, Darmstadt, Germany]). The expression of A3G, A3H and tubulin were detected by using anti-hA3H (1:1,000) [35], anti-hA3G (1:10,000, NIH AIDSREAGENTS, cat. 9906) [69] and anti-tubulin (1:4,000 dilution, clone B5-1-2; Sigma-Aldrich, Taufkirchen, Germany) antibodies, respectively.

### Phylogenetic analysis

The primate A3H sequences were obtained from GenBank, the accession numbers are: EU861357, EU861358, EU861359, EU861360, EU861361, DQ408606 and DQ507277. CpzA3H SNPs were described in this study. The A3H sequences were aligned using the ClustalW in Mega 7 software. The phylogenetic analysis was performed in Mega 7 by using bootstrap neighbor joining method. Test parameters were estimated using 500 bootstrap replicates.

### Model structure

To analyze the interaction surface between SIVcpz Vif and hA3H hapII, the structure of SIVcpz Vif was modeled using HIV-1 Vif (4N9F) [70] as template by using SWISS-MODEL online server (http://www.swissmodel.expasy.org/). The recent crystal structure of hA3H hapII (6B0B) was also used to model the structure of cpzA3H. The SIVcpz Vif and hA3H hapII co-structure was modeled based on the recent HIV-1 Vif-A3H interaction surface analysis [49]. The graphical visualization was constructed using PyMOL (PyMOL Molecular Graphics System, version 1.5.0.4; Schrödinger, Portland, OR).

### Statistical analysis

Data are represented as the mean with SD in all bar diagrams. Statistically significant differences between two groups were analyzed using the unpaired Student’s t-test with GraphPad Prism version 5 (GraphPad software, San Diego, CA, USA). A minimum p value of 0.05 was considered as statistically significant: P value < 0.001 extremely significant (***), 0.001 to 0.01 very significant (**), 0.01 to 0.05 significant (*), >0.05 not significant (ns).

## Supporting information

S1 Fig Schematic genome structure of SIVcpz*Ptt*MB897 and SIVcpz*Pts*TAN1. The restriction sites used for construction of nanoluciferase (NLuc) reporter viruses are shown. Stop codons were inserted in *vif* at positions for coding of amino acid 40 and 44.
(TIF)

S2 Fig **Detection of A3 expression by immunoblots** (**a, b, c**): 293T cells were transfected with 30 ng hA3s or 200 ng cpzA3s expression plasmids. Two days post-transfection, cell lysates were used to detect the expression of A3s by two different anti-HA antibodies. Tubulin served as a loading control. (**d**) SIVcpz*Ptt*MB897 or SIVcpz*Pts*TAN1 wild type or delta *vif* reporter viruses were produced in 293T cells in the presence of cpzA3H or hA3H hapII, pcDNA3.1(+) was used as control (vector). Two days post-transfection, cpzA3H and hA3H hapII in cell lysates and viral particles were detected by anti-HA antibody. Viral capsid (p24) was detected by anti-p24 antibody. Tubulin served as a loading control. VLP: Viral Like Particle.
(TIF)

S3 Fig (**a**) Characterization of SupT11-vetor-hCCR5 or SupT11-hA3H hapII-hCCR5 cells for expression of hA3H hapII using immunoblots of cell lysates and anti-hA3H antibody. Tubulin served as a loading control and (**b**) for expression of CCR5 and CD4 by flow cytometry. Cells were stained by α-hCCR5 FITC, or α-hCD4 PE mouse IgG1_k_ separately. The mouse IgG1/RPE isopeptidase was used as negative antibody control for CD4 staining. (**c**) SupT11-vetor-hCCR5 or SupT11-hA3H hapII-hCCR5 cells were infected with 50 ng RT activity of SIVcpz*Pts*TAN1, SIVcpz*Ptt*MB897 or SIVcpz*Ptt*MB897_EN-PH (47EN48 replaced by 47PH48 in Vif open reading frame), respectively, and culture supernatants were collected each second day and quantified by the RT assay.
(TIF)

S4 Fig **Structural superimposition of cpzA3H** (**a**) The recent crystal structure of hA3H hapII (6B0B) was used to model the structure of cpzA3H. The SNPs of cpzA3H identified in this study were shown. (**b**) The potential SIVcpz/HIV-1 Vif interaction sites in helix-3 and helix-4 of cpzA3H (green) are shown.
(TIF)
