# Supplementary figures and images for: Stably expressed APOBEC3H forms a barrier for cross-species transmission of simian immunodeficiency virus of chimpanzee to humans
Source: PLoS Pathog. 2017 Dec 21;13(12):e1006746. doi: 10.1371/journal.ppat.1006746 (PMC5739507; doi:10.1371/journal.ppat.1006746)

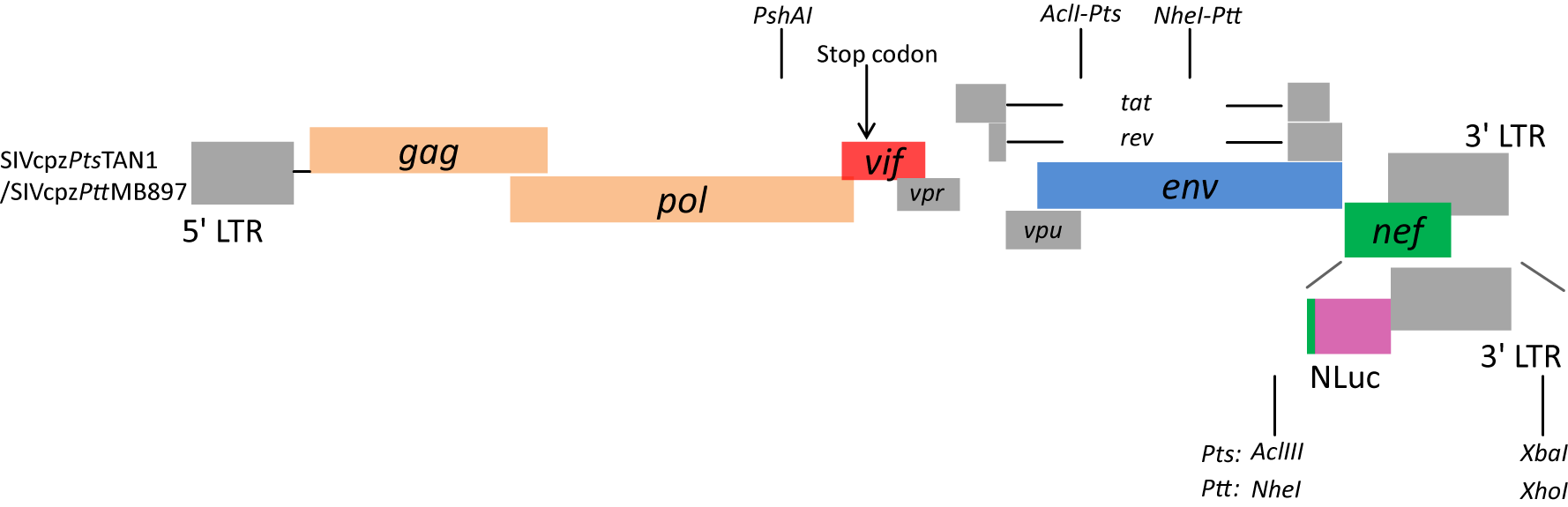

Supplement: S1 Fig — The restriction sites used for construction of nanoluciferase (NLuc) reporter viruses are shown. Stop codons were inserted in vif at positions for coding of amino acid 40 and 44. (TIF) [file ppat.1006746.s001.tif]

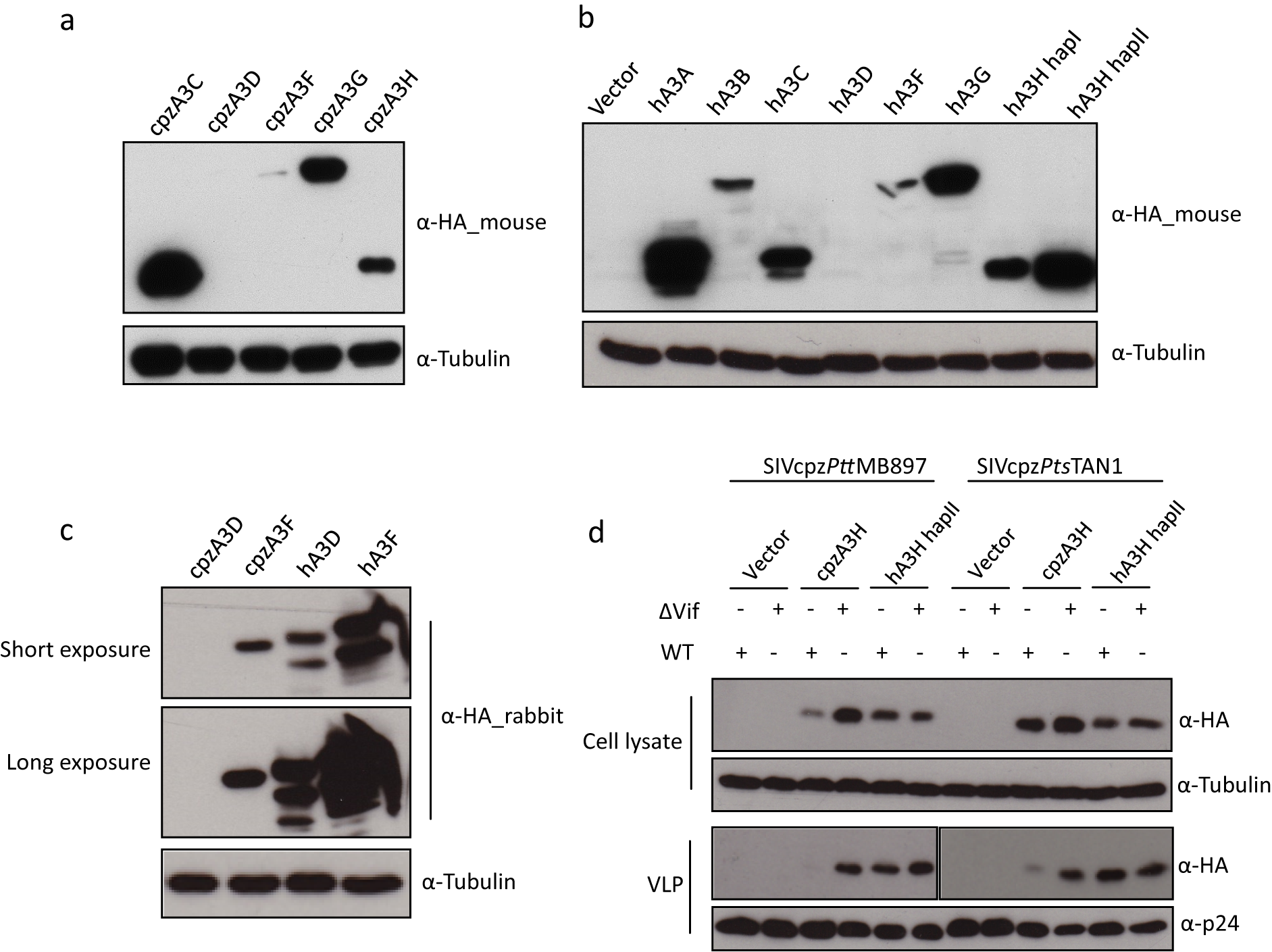

Supplement: S2 Fig — Detection of A3 expression by immunoblots (a, b, c): 293T cells were transfected with 30 ng hA3s or 200 ng cpzA3s expression plasmids. Two days post-transfection, cell lysates were used to detect the expression of A3s by two different anti-HA antibodies. Tubulin served as a loading control. (d) SIVcpzPttMB897 or SIVcpzPtsTAN1 wild type or delta vif reporter viruses were produced in 293T cells in the presence of cpzA3H or hA3H hapII, pcDNA3.1(+) was used as control (vector). Two days post-transfection, cpzA3H and hA3H hapII in cell lysates and viral particles were detected by anti-HA antibody. Viral capsid (p24) was detected by anti-p24 antibody. Tubulin served as a loading control. VLP: Viral Like Particle. (TIF) [file ppat.1006746.s002.tif]

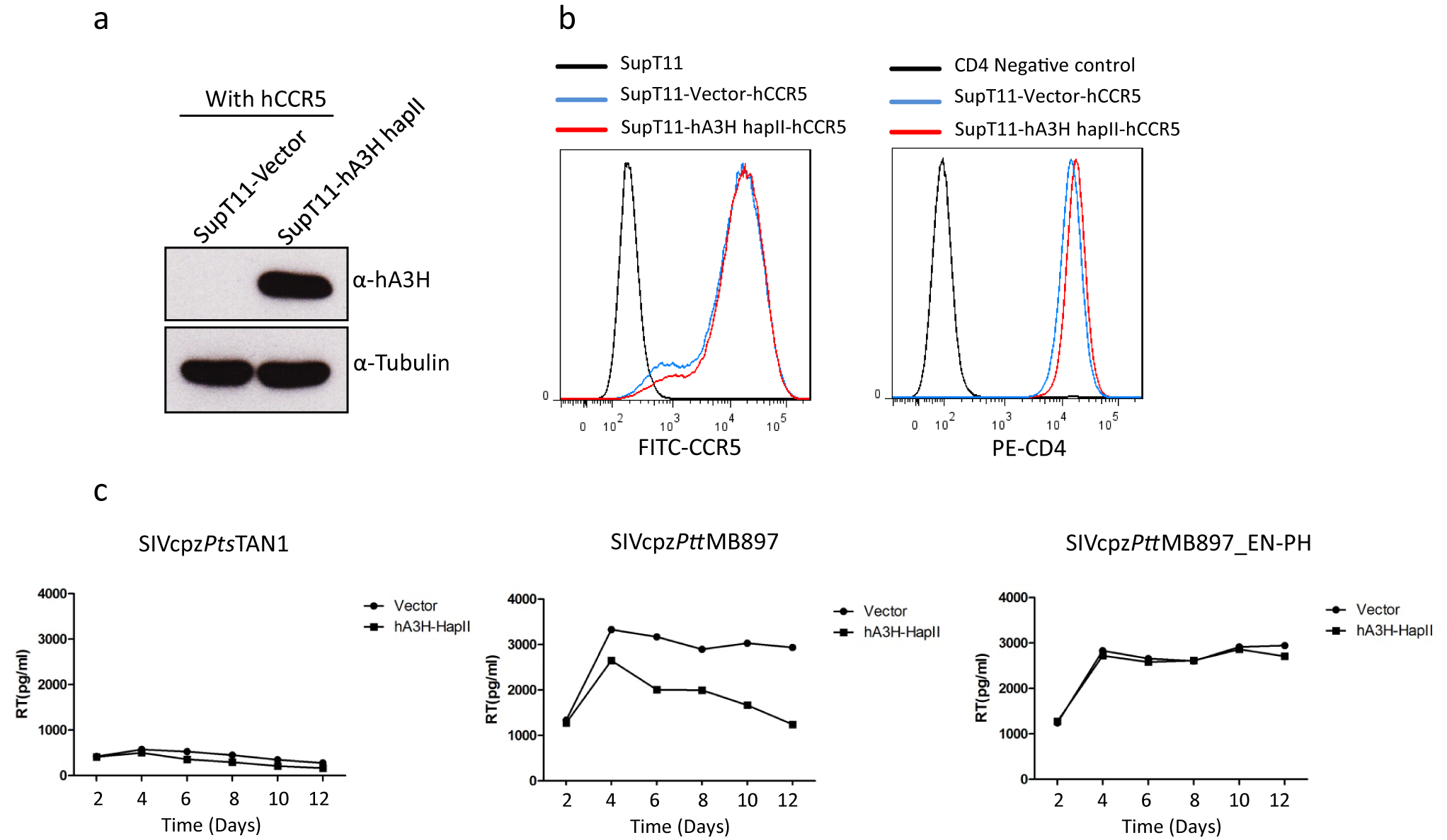

Supplement: S3 Fig — (a) Characterization of SupT11-vetor-hCCR5 or SupT11-hA3H hapII-hCCR5 cells for expression of hA3H hapII using immunoblots of cell lysates and anti-hA3H antibody. Tubulin served as a loading control and (b) for expression of CCR5 and CD4 by flow cytometry. Cells were stained by α-hCCR5 FITC, or α-hCD4 PE mouse IgG1k separately. The mouse IgG1/RPE isopeptidase was used as negative antibody control for CD4 staining. (c) SupT11-vetor-hCCR5 or SupT11-hA3H hapII-hCCR5 cells were infected with 50 ng RT activity of SIVcpzPtsTAN1, SIVcpzPttMB897 or SIVcpzPttMB897_EN-PH (47EN48 replaced by 47PH48 in Vif open reading frame), respectively, and culture supernatants were collected each second day and quantified by the RT assay. (TIF) [file ppat.1006746.s003.tif]

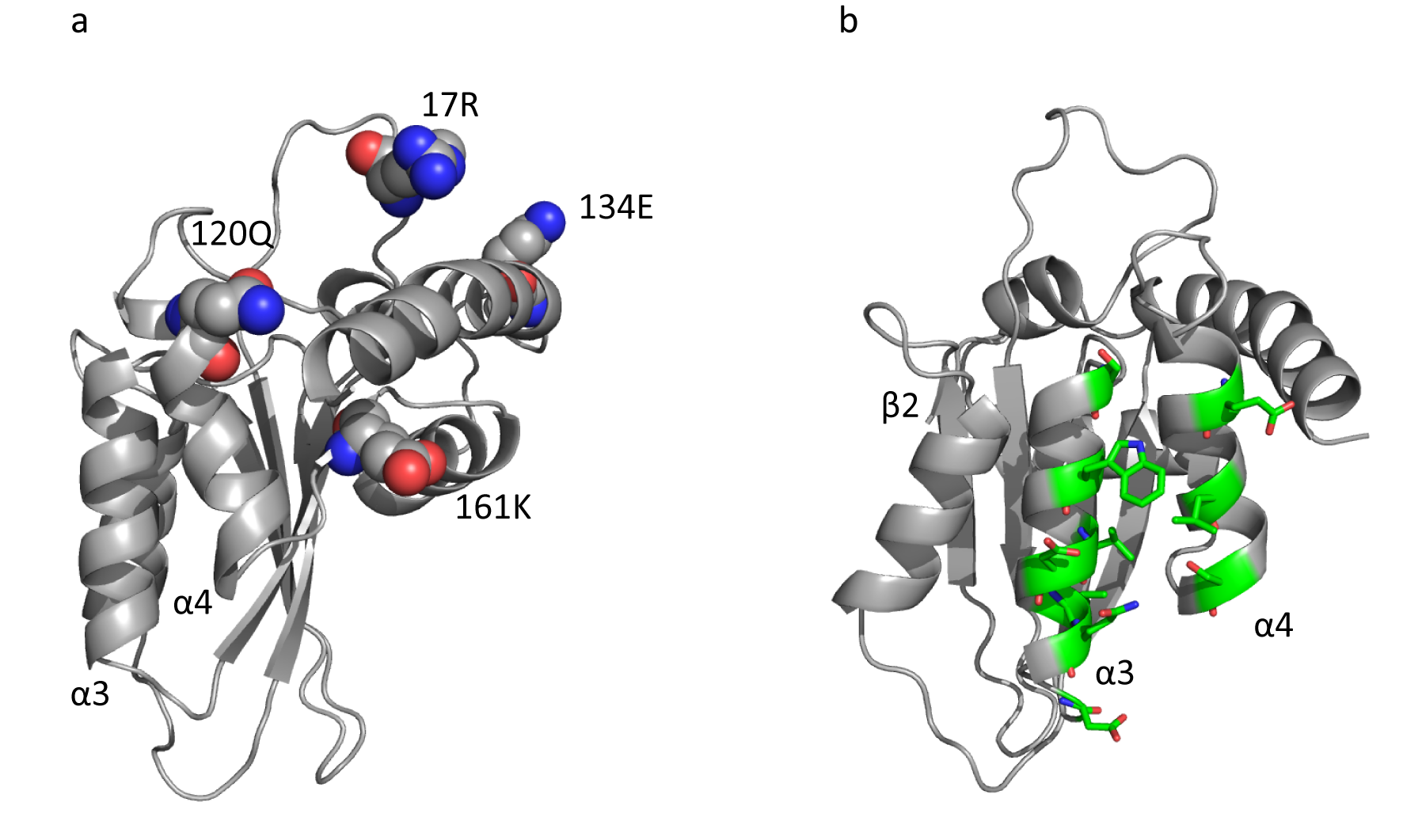

Supplement: S4 Fig — Structural superimposition of cpzA3H (a) The recent crystal structure of hA3H hapII (6B0B) was used to model the structure of cpzA3H. The SNPs of cpzA3H identified in this study were shown. (b) The potential SIVcpz/HIV-1 Vif interaction sites in helix-3 and helix-4 of cpzA3H (green) are shown. (TIF) [file ppat.1006746.s004.tif]
